# Adhesion- and stress-related adaptation of glioma radiochemoresistance is circumvented by β1 integrin/JNK co-targeting

**DOI:** 10.18632/oncotarget.17480

**Published:** 2017-04-27

**Authors:** Anne Vehlow, Erik Klapproth, Katja Storch, Ellen Dickreuter, Michael Seifert, Antje Dietrich, Rebecca Bütof, Achim Temme, Nils Cordes

**Affiliations:** ^1^ OncoRay, National Center for Radiation Research in Oncology, Faculty of Medicine and University Hospital Carl Gustav Carus, Technische Universität Dresden, Helmholtz-Zentrum Dresden, Rossendorf, PF 41, 01307, Dresden, Germany; ^2^ Helmholtz-Zentrum Dresden, Rossendorf, Institute of Radiopharmaceutical Cancer Research, 01328, Dresden, Germany; ^3^ Helmholtz-Zentrum Dresden, Rossendorf, Institute of Radiooncology, OncoRay, 01328, Dresden, Germany; ^4^ Institute for Medical Informatics and Biometry, Faculty of Medicine Carl Gustav Carus, Technische Universität Dresden, 01307, Dresden, Germany; ^5^ National Center for Tumor Diseases (NCT), Partner Site Dresden, 69192, Heidelberg, Germany; ^6^ Department of Radiotherapy and Radiation Oncology, Faculty of Medicine and University Hospital Carl Gustav Carus, Technische Universität Dresden, PF 50, 01307, Dresden, Germany; ^7^ Department of Neurosurgery, Section of Experimental Neurosurgery/Tumor Immunology, Faculty of Medicine and University Hospital Carl Gustav Carus, Technische Universität Dresden, 01307, Dresden, Germany; ^8^ German Cancer Consortium (DKTK), Parter Site Dresden, 69192, Heidelberg, Germany; ^9^ German Cancer Research Center (DKFZ), 69192, Heidelberg, Germany; ^10^ Current address: National Center for Tumor Diseases (NCT), Partner Site Dresden, 69192, Heidelberg, Germany; ^11^ Current address: German Cancer Consortium (DKTK), Parter Site Dresden, and German Cancer Research Center (DKFZ), 69192, Heidelberg, Germany

**Keywords:** β1 integrin, JNK, radiochemoresistance, GBM stem-like cells, orthotopic GBM mouse model

## Abstract

Resistance of cancer stem-like and cancer tumor bulk cells to radiochemotherapy and destructive infiltration of the brain fundamentally influence the treatment efficiency to cure of patients suffering from Glioblastoma (GBM). The interplay of adhesion and stress-related signaling and activation of bypass cascades that counteract therapeutic approaches remain to be identified in GBM cells. We here show that combined inhibition of the adhesion receptor β1 integrin and the stress-mediator c-Jun N-terminal kinase (JNK) induces radiosensitization and blocks invasion in stem-like and patient-derived GBM cultures as well as in GBM cell lines. *In vivo*, this treatment approach not only significantly delays tumor growth but also increases median survival of orthotopic, radiochemotherapy-treated GBM mice. Both, *in vitro* and *in vivo*, effects seen with β1 integrin/JNK co-inhibition are superior to the monotherapy. Mechanistically, the *in vitro* radiosensitization provoked by β1 integrin/JNK targeting is caused by defective DNA repair associated with chromatin changes, enhanced ATM phosphorylation and prolonged G2/M cell cycle arrest. Our findings identify a β1 integrin/JNK co-dependent bypass signaling for GBM therapy resistance, which might be therapeutically exploitable.

## INTRODUCTION

Owing to the radiochemoresistant and infiltrative nature of Glioblastoma (GBM) [1–4], the prognosis of GBM patients is poor with an estimated five year overall survival of less than five percent [5, 6]. Despite advances in multimodal therapy, GBM remains a disease of substantial unmet need and it has become increasingly clear that both key genetic mutations and microenvironmental cues drive intrinsic and acquired therapy resistance [2, 7–9]. The application of molecular-targeted agents offers a wealth of therapeutic options but similarly challenges the great potential of tumor cells to utilize effective adaptation mechanisms [10, 11]. We have recently published such an example in head and neck cancers, where the dual inhibition of integrin/receptor tyrosine kinase (RTK) effectively deactivated prosurvival bypass signaling and induced stronger radiosensitization and tumor cure *in vivo* [12, 13]. Thus, understanding the molecular mechanisms that drive adaptation to therapy may lead to individualized multi-targeting approaches concomitant to conventional radiochemotherapy.

Among the plethora of candidates activated by cellular stress such as radiotherapy are c-Jun N-terminal kinases (JNK1, JNK2, JNK3) [14]. In cancer, JNK promote proliferation, survival, motility and transcription factor phosphorylation like c-Jun by signal transduction and cytoplasmic-to-nuclear translocation [14, 15]. JNK's aberrant phosphorylation and activity in human GBM emphasizes a critical involvement in prosurvival signaling that facilitates tumor progression through regulation of self-renewal and tumor-initiating properties of GBM stem-like cells and their resistance to the standard therapeutic Temozolomide (TMZ) by regulating MGMT expression [16–21]. However, the role and association of JNK with microenvironmental factors leading to GBM radioresistance and invasion remains unclear.

Linking to the microenvironment, integrin receptors critically mediate prosurvival and proinvasive signaling upon cell adhesion to extracellular matrix (ECM) [22, 23]. Following exposure to irradiation, integrins are upregulated in GBM cells and contribute to cell adhesion-mediated radioresistance [24, 25]. Furthermore, several of the 8 beta and 18 alpha integrin subunits are overexpressed in GBM and a multitude of human malignancies, and are regarded as potential cancer targets owing to their role in tumor progression and metastasis [4, 26–28]. After the surprising failure of Cilengitide as αvβ3/β5 integrin-antagonistic GBM therapeutic in the CENTRIC phase III clinical trial [29], alternative strategies focusing on the versatile β1 integrin subunit are currently under intense investigation to identify their radiochemosensitizing and anti-migratory potential [4, 28, 30–32]. Interestingly, β1 integrin and JNK are linked upon irradiation in an entity-dependent manner [33–35], but whether the crosstalk of adhesion and stress-related signaling is implicated in GBM adaptation, radioresistance and invasion has not been investigated.

The presented study exploited the potential contextual synthetic lethal adaptation arising from β1 integrin and JNK cooperation by simultaneous inhibition of these two target molecules in GBM stem-like and patient-derived GBM cell cultures as well as GBM cell lines. We found dual β1 integrin/JNK targeting to be superior to monotherapy, which translated into radiosensitization and blocked cell invasion. Strikingly, β1 integrin/JNK inhibition concomitantly applied to radiochemotherapy demonstrated significant tumor growth delay and increased median survival of mice bearing orthotopic GBM. Mechanistically, the radiosensitization by β1 integrin/JNK co-inhibition was entailed by chromatin changes, enhanced DNA double strand breaks, associated ATM hyperphosphorylation and a prolonged G2/M cell cycle arrest.

## RESULTS

### β1 integrin/JNK co-targeting sensitizes GBM cells to radiotherapy

**Figure 1:**
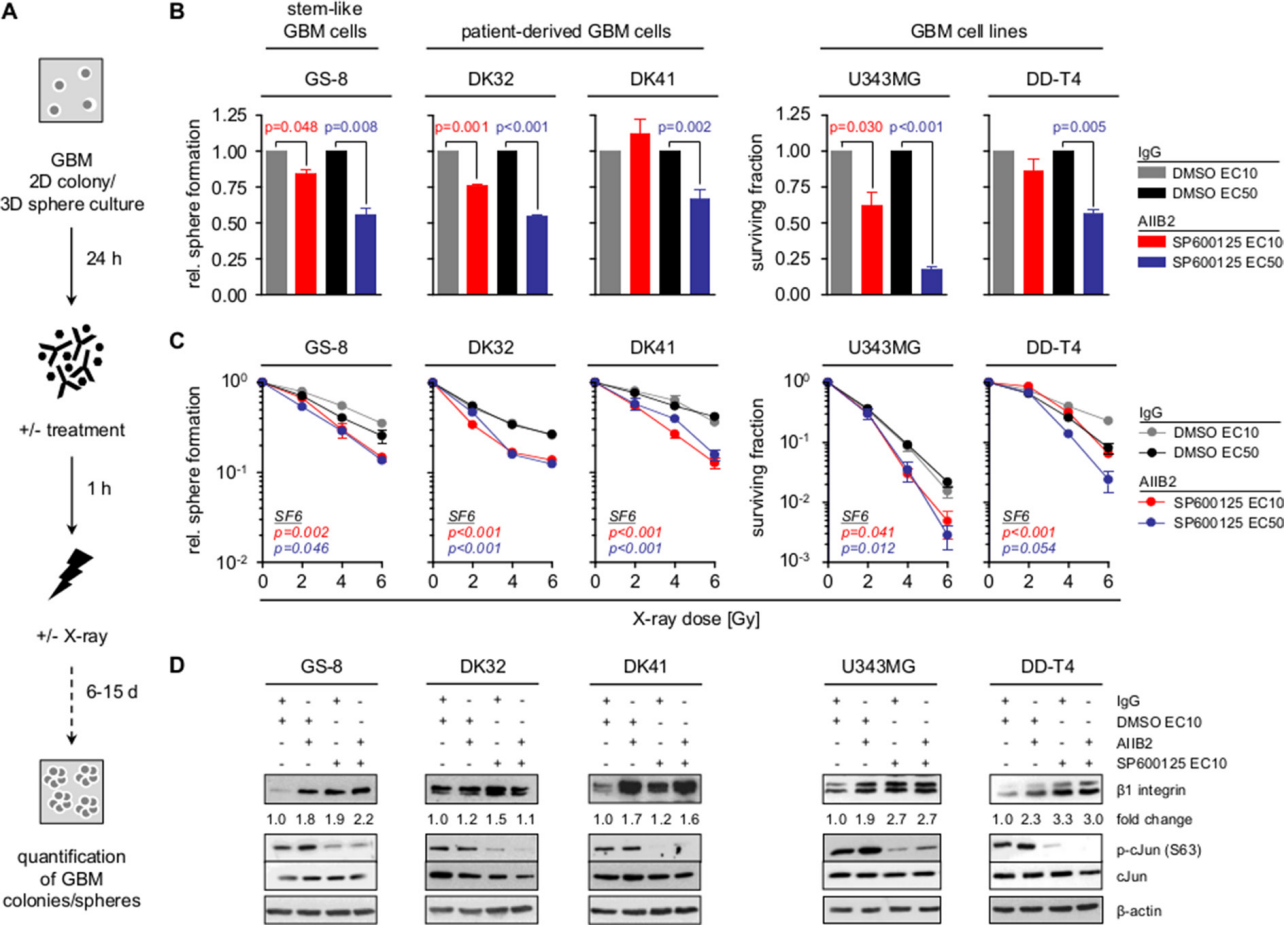
Co-targeting of β1 integrin and JNK sensitizes GBM cells to radiotherapy (**A**) Workflow of GBM sphere formation and clonogenic survival assay. (**B**) Relative sphere formation and basal surviving fraction of GBM stem-like cells (GS-8), patient-derived GBM cultures (DK32, DK41) and GBM cell lines (U343MG, DD-T4) upon treatment with AIIB2/SP600125 (EC10, EC50) or controls (IgG, DMSO). (**C**) Relative sphere formation and clonogenic survival upon treatment with AIIB2/SP600125 (EC10, EC50) and X-ray irradiation (2, 4, 6 Gy) (controls are IgG and DMSO). (B, C) Results are mean +/− SEM (*n* = 3–4, *t*-test). (**D**) Western blot analysis of β1 integrin, phospho-cJun (S63), cJun and β-actin of whole cell lysates from indicated GBM cells treated as indicated with AIIB2 (10 μg/ml), SP600125 (EC10), IgG control (10 μg/ml) and DMSO. Fold change is calculated by normalization to β-actin and IgG/DMSO controls according to representative blots.

As β1 integrin and JNK signaling are critically involved in GBM cell survival [19, 20, 24, 30] and the radiosensitizing potential of their specific targeting unclear, we tested sphere forming capacity and clonogenicity of GBM stem-like cells (GS-8; MGMT positive and TMZ resistant), patient-derived GBM cell cultures (DK32, DK41) (PDC) and GBM cell lines (U343MG, DD-T4) treated either with the β1 integrin-specific inhibitory antibody AIIB2, the JNK inhibitor SP600125 (JNKi) or the AIIB2/JNKi combination (Figure 1, Supplementary Figures 1 and 2). While JNKi mediated cytotoxicity concentration-dependently in all GBM cell populations, neither the 10% effective concentration (EC10) nor the EC50 of JNKi radiosensitized GBM cells (Supplementary Figure 1A–1C, all *p*-values in Supplementary Table 1). Similarly, siRNA-mediated depletion of JNK1 and JNK2 isoforms reduced basal survival but did not induce radiosensitization of U343MG cells (Supplementary Figure 3A–3C). Despite the radiosensitizing effects of AIIB2 in head and neck cancer [12, 35], β1 integrin targeting failed to affect basal and radiation survival of all GBM cells tested (Supplementary Figure 2A–2B). Based on the reported connection of β1 integrin and JNK signaling in other tumor entities [33–35], as well as β1 integrin upregulation and JNK hyperphosphorylation in GBM [4, 16], we next inhibited both molecules simultaneously. Intriguingly, co-targeting of β1 integrin/JNK mediated cytotoxicity and additive radiosensitization in GBM stem-like cells and PDC as well as GBM cell lines relative to controls and superior to monotherapies (Figure 1A–1C, Supplementary Table 2). We discovered increases in β1 integrin expression upon exposure to AIIB2, JNKi or the combination (Figure 1D). As a β1 integrin overexpression is well known in GBM (Supplementary Figure 4), we are tempted to speculate that this induction of β1 integrin expression might be utilized, in addition to yet unkown processes, by GBM cells to facilitate cell survival in response to therapeutics. Taken together our data evidently show that dual blockage of β1 integrin and JNK is required for GBM cell radiosensitization.

### Combined targeting of β1 integrin and JNK blocks GBM invasion

**Figure 2:**
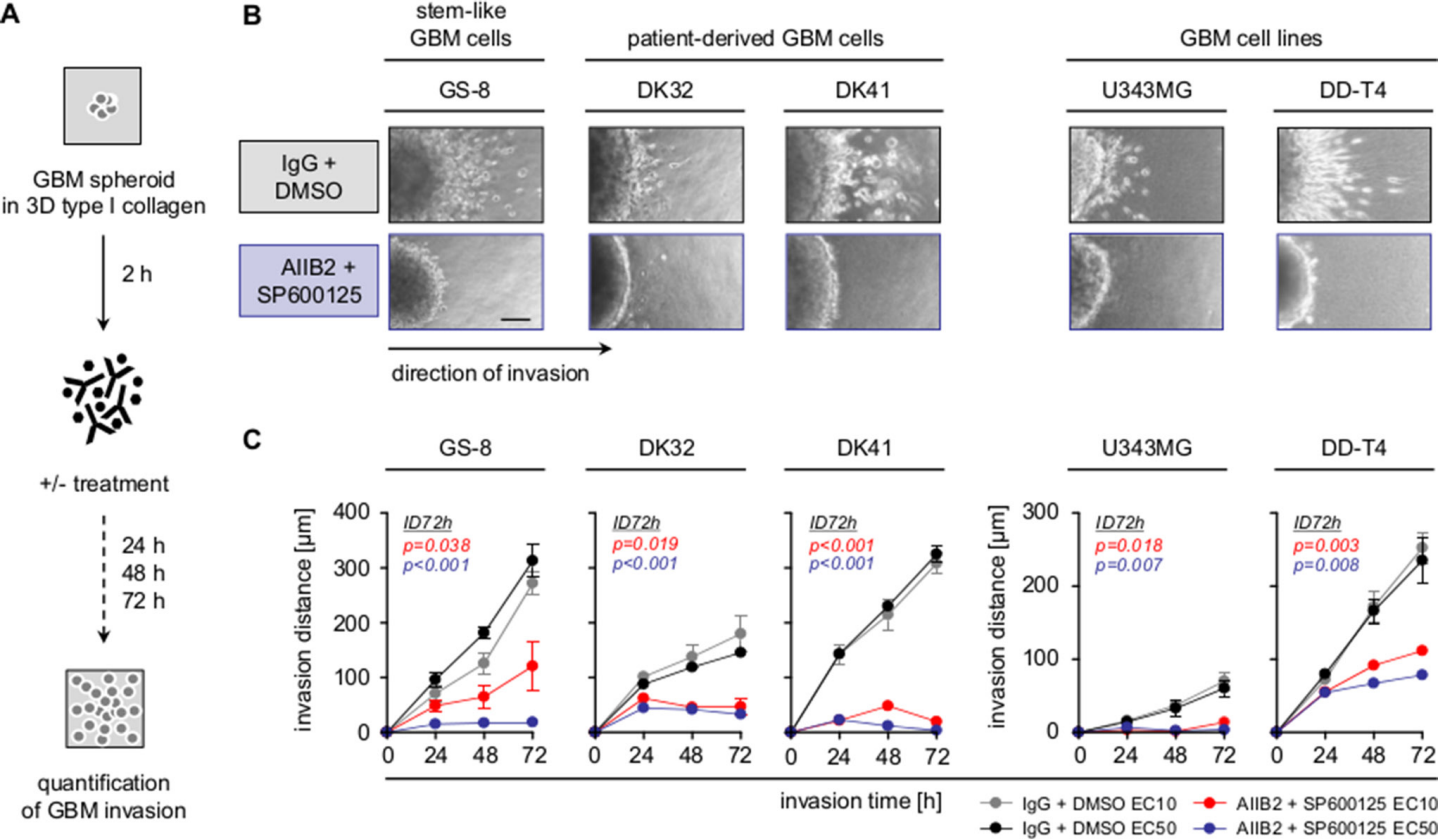
Combined β1 integrin/JNK targeting blocks GBM cell invasion (**A**) Work flow of 3D invasion assays. (**B**) Representative images of indicated GBM cells invading into 3D type I collagen 72 h after treatment with AIIB2/SP600125 (EC50) or control IgG/DMSO. Scale bar, 50 μm. (**C**) Invasion distance of indicated treated GBM cell populations. Results are mean +/− SEM (*n* = 3, *t*-test).

In addition to radioresistance, the infiltrative nature of GBM remains a key challenge for GBM therapy and may be associated with type I collagen, one of the elevated, invasion and stemness associated ECM proteins in GBM (Supplementary Figure 4) [4, 36]. Therefore, we tested the effect of a combined β1 integrin/JNK blockage on 3D collagen invasion of the different GBM cell populations (Figure 2A). Most remarkably, dual targeting led to a strong and significant reduction of the invasion capacity of GBM stem-like cells, PDCs and GBM cell lines (Figure 2B–2C). Furthermore and in line with matrix metalloproteinase (MMP)-dependent ECM degradation for mesenchymal invasion [4], the secretion of MMP2 and MMP9 was severely diminished upon β1 integrin/JNK inhibition (Supplementary Figure 5A–5B). These observations demonstrate that the dual blockage of β1 integrin/JNK hampers GBM invasion on top of radiosensitization.

### Simultaneous β1 integrin/JNK inhibition delays tumor growth and prolongs survival in combination with radiochemotherapy *in vivo*

**Figure 3:**
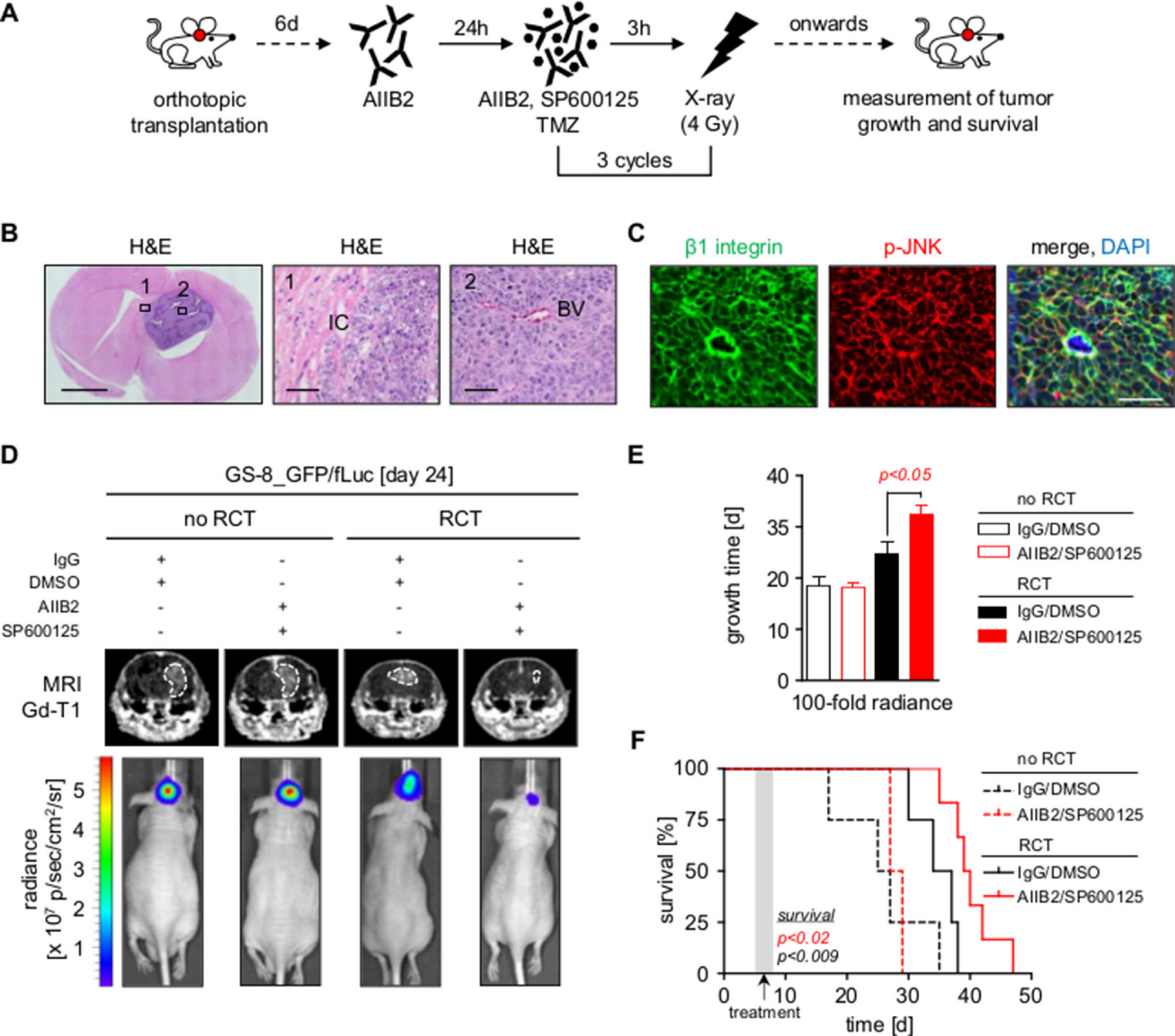
Dual inhibition of β1 integrin and JNK delays tumor growth and prolongs survival in combination with radiochemotherapy (**A**) Treatment scheme of mice bearing tumors of GS-8_GFP/fLuc stem-like cells. (**B**) Left panel shows representative image of a mouse brain with GS-8_GFP/fLuc tumor. Scale bar, 2 mm. Region 1 and 2 corresponding to middle and right panel magnifications show invading tumor cells (IC) and blood vessel (BV) of GS-8_GFP/fLuc tumors, respectively. Scale bar, 100 μm. (**C**) Representative images from GS-8_GFP/fLuc tumors showing β1 integrin (green), phospho-JNK (T183/Y185) (red) or merge with nuclei (DAPI, blue). Scale bar, 100 μm. (**D**) Gadolinium-enhanced (Gd) T1-weighted magnetic resonance images (MRI) from mice bearing GS-8_GFP/fLuc tumors 24 days after transplantation and β1 integrin/JNK inhibition without or in combination with radiochemotherapy (RCT). Dashed lines delineate tumors. Lower images show luminescence analyses of representative GS-8_GFP/fLuc tumors 24 days after transplantation and indicated treatment. (**E**) GS-8_GFP/fLuc tumor growth time to 100 fold radiance after indicated treatment. Data are mean +/− SEM (6 - 8 mice per group, one-way ANOVA). (**F**) Survival of GS-8_GFP/fLuc mice treated as indicated. Kaplan Meier analysis includes 6 mice IgG/DMSO, 8 mice AIIB2/SP600125, 6 mice IgG/DMSO+RCT, 6 mice AIIB2/SP600125+RCT (two-sided log rank test, *p* < 0.009: IgG/DMSO+RCT vs IgG/DMSO, *p* < 0.02: AIIB2/SP600125+RCT vs IgG/DMSO+RCT).

We next assessed the therapeutic relevance of dual β1 integrin/JNK targeting in an orthotopic mouse model using GS-8 GBM stem-like cells expressing EGFP and firefly luciferase (GS-8_GFP/fLuc), which had engrafted and recruited vasculature at the time of treatment (Figure 3A–3C, data not shown). It was important to us to focus on the impact of an AIIB2/JNKi treatment in addition to the standard of care for GBM applying radiotherapy plus TMZ, as molecular-targeted agents will, at least in the near future, be adjuvantly administered. Magnetic resonance imaging (MRI) and luminescence of mice receiving AIIB2/JNKi concomitantly administered to conventional radiotherapy/TMZ (RCT) showed significantly delayed tumor growth relative to mice receiving either AIIB2/JNKi or RCT alone (Figure 3D–3E). Additionally, AIIB2/JNKi/RCT also translated into a significant improvement of median overall survival relative to other treatment groups (Figure 3F) without effects on mouse weight or behavior during the tumor growth period (data not shown). Treatment of mice with either AIIB2 or JNKi alone with or without RCT did not affect tumor growth or survival (Supplementary Figure 6A–6B). Thus, our observations show simultaneous β1 integrin/JNK targeting in combination with RCT to be superior to RCT, AIIB2 and JNKi alone in terms of tumor growth delay and overall survival in an orthotopic GBM model.

### β1 integrin and JNK deactivation differentially impacts on various regulatory networks

**Figure 4:**
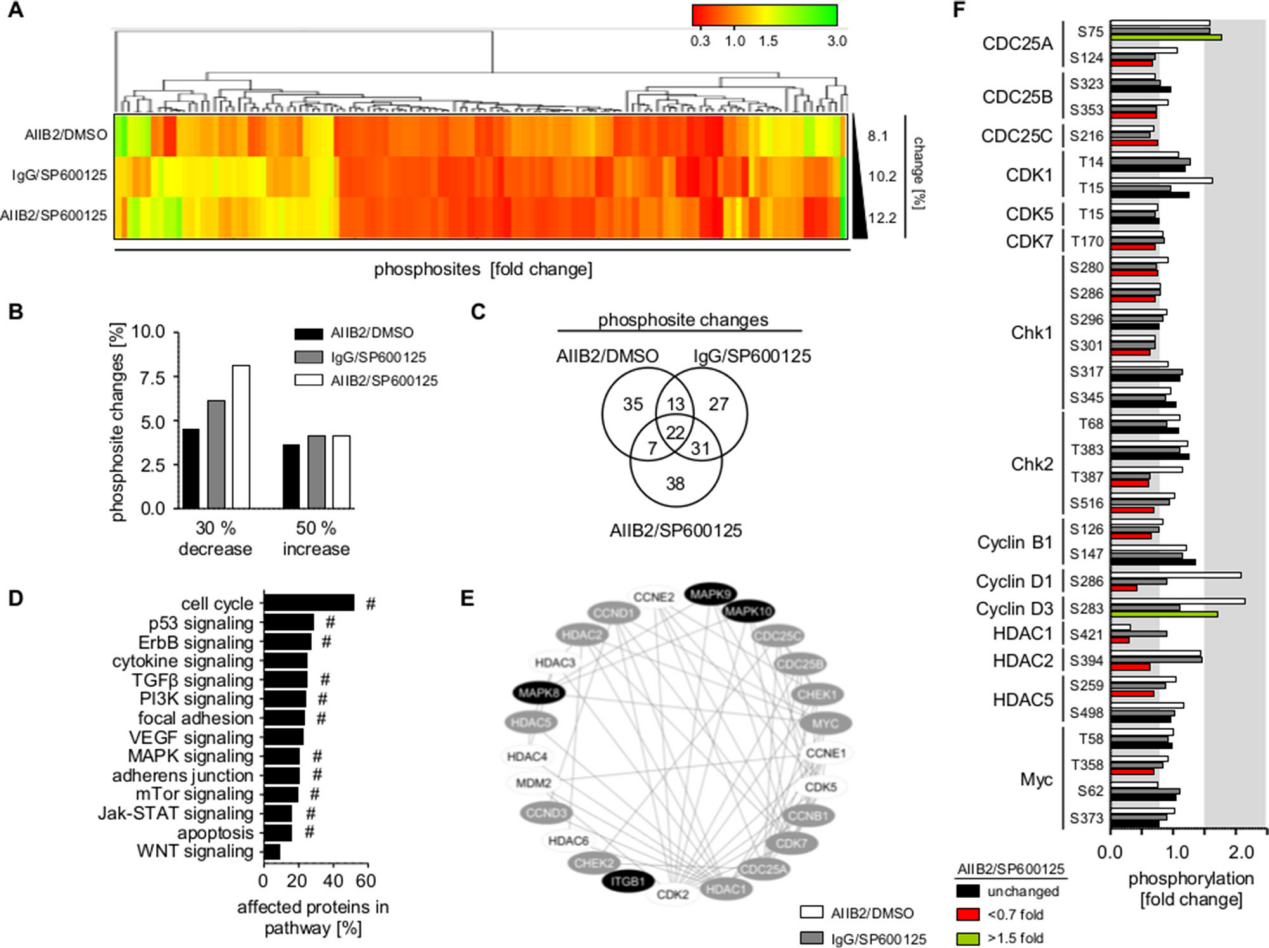
β1 integrin/JNK co-targeting interferes with cell cycle regulatory networks (**A**) Hierarchical clustering of altered phosphorylation sites (30% decrease, 50% increase, fold change) from phosphoproteome analysis of U343MG cells 1 h after indicated treatment with AIIB2, JNKi or AIIB2/JNKi (EC10) normalized to controls (percentage of phosphorylation site changes is shown on the right). (**B**) Percentage of phospho-sites showing 30% decreased and 50% increased phosphorylation upon indicated treatment among the 606 phosphorylations sited investigated in the phosphoproteome analysis. (**C**) Venn diagram analysis of altered phosphosites from (A). (**D**) Functional classification of altered proteins after treatment with AIIB2/SP600125 (EC10) in comparison to the IgG/DMSO controls. Enrichment of genes in signaling pathway: ^#^*p* < 0.01 (Fisher's exact test). (**E**) Cytoscape-based molecular interaction network of β1 integrin and JNK isoforms (black) with altered (grey) and not altered (white) cell cycle associated proteins. (**F**) Fold change in phosphorylation of cell cycle associated proteins. Threshold of phosphosite changes (30% decrease and 50% increase) is marked in grey.

Next, we addressed the underlying mechanisms causative for radiosensitization by β1 integrin/JNK inhibition. Broad insight into signal transduction was gained by phosphoproteome analysis of 606 phosphosites from 342 proteins (Supplementary Table 3) in U343MG cells treated with AIIB2, JNKi or AIIB2/JNKi. Within the threshold of 30% reduced or 50% increased phosphorylation, simultaneous AIIB2/JNKi application resulted in higher percentage of phosphosite changes (∼12%) as compared to monotherapies (∼8–10%) (Figure 4A–4B; Supplementary Tables 3 and 4). Venn diagram analysis of the altered phosphosites upon single AIIB2, single JNKi or dual AIIB2/JNK treatment showed only partial overlap (35 out of 135), indicating convergence of β1 integrin and JNK signaling at some but not all regulatory nodes (Figure 4C, Supplementary Figure 7B–7C). Furthermore, functional classification of proteins with phosphosite changes after AIIB2/JNKi therapy suggested that cell cycle regulation followed by p53 and ErbB signaling is predominantly affected and linked to β1 integrin and JNK in molecular interaction networks (Figure 4D–4F, Supplementary Figure 7A).

### HDAC function and DNA repair are co-regulated by β1 integrin and JNK

**Figure 5:**
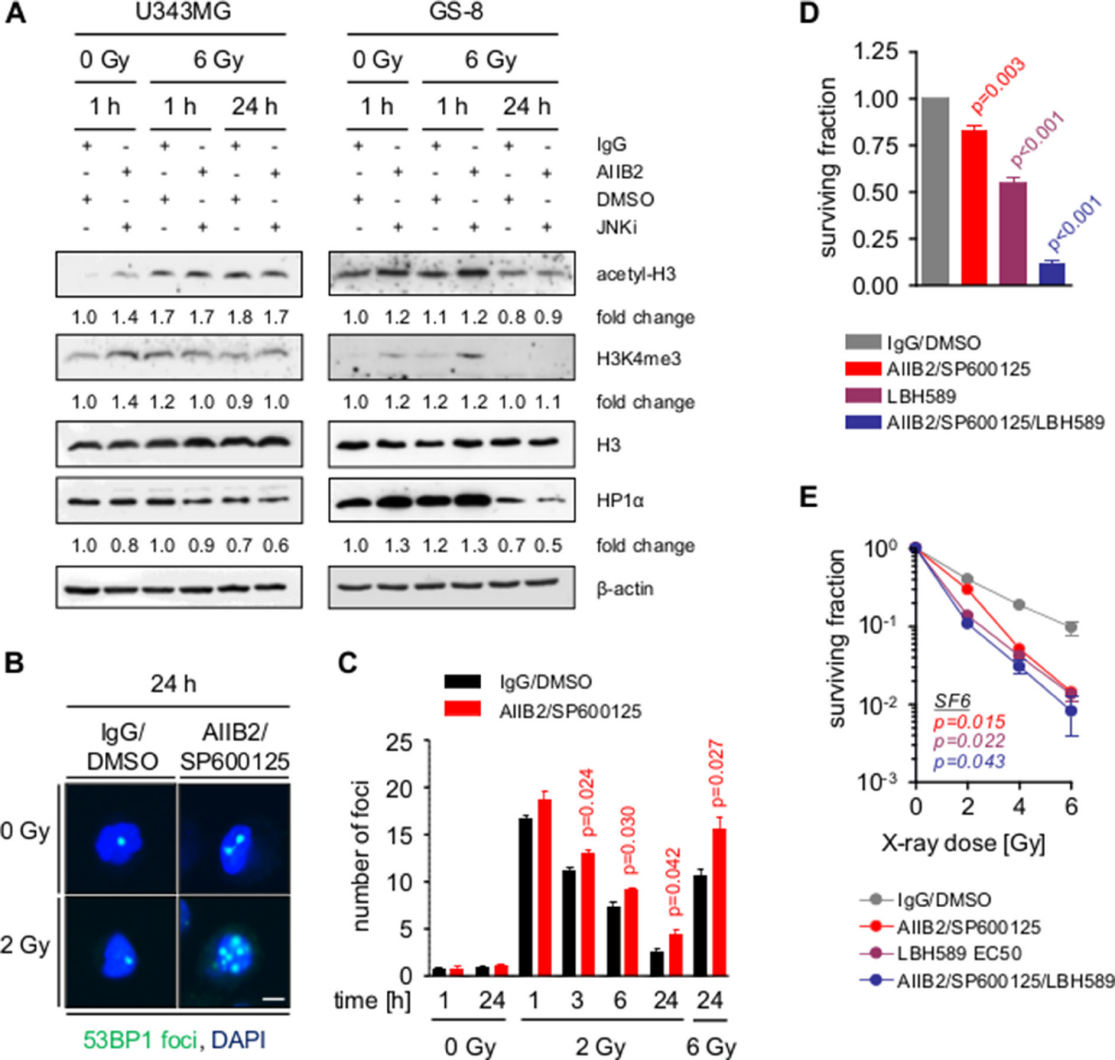
Block of β1 integrin and JNK signaling enhances radiation induced DSB by chromatin modification (**A**) Western blot analysis of indicated proteins from whole cell lysates of U343MG and GS-8 cells treated with AIIB2/SP600125 (EC10) or control IgG/DMSO without and with X-ray irradiation (6 Gy). Fold changes are calculated by normalization to β-actin and IgG/DMSO controls according to representative blots. (**B**) Immunofluorescence analysis of nuclei with 53BP1-positive foci after indicated treatments. Scale bar, 10 μm. (**C**) Quantification of the number of DSB per cell at the indicated time points after treatment with AIIB2/SP600125 (EC10) or control IgG/DMSO without and with X-ray irradiation. (**D**) Basal surviving fraction of U343MG cells upon treatment with AIIB2/SP600125 (EC10), LBH589 (EC50) or a combination thereof compared to control treatment (IgG/DMSO). (**E**) Clonogenic radiation survival of U343MG cells treated as described in (H). (C–E) Results are mean +/− SEM (*n* = 3, *t*-test).

Among the altered proteins found in the phosphoproteome analysis were histone deacetylases (e.g. HDAC1, HDAC2, HDAC5) (Figure 4E–4F), which are well known to contribute to therapy resistance and DNA damage repair through altering chromatin organization [37–40]. Indicative of chromatin decondensation at the time of irradiation, we discovered a 1.4-fold increase in both histone 3 (H3) acetylation and trimethyl-K4 (K4me3) levels 1 h after combined AIIB2/JNKi exposure, which was accompanied by a reduction of the HP1α level in U343MG and GS-8 GBM cells (Figure 5A). In line with these results, AIIB2/JNKi treatment caused significantly compromised DSB repair at early and late time points after 2 and 6 Gy X-ray irradiation (Figure 5B–5C, Supplementary Figure 8). To provide the contextual interlacing of β1 integrin/JNK and HDAC function, we assessed basal and clonogenic radiation survival upon single treatment with LBH589 (HDAC inhibitor, HDACi) or a combination thereof with AIIB2/JNKi. Single addition of the HDACi resulted in a dose-dependent decrease of U343MG cell survival and radiosensitization (Supplementary Figure 9). Intriguingly, the triple treatment with HDACi/AIIB2/JNKi further decreased basal cell survival, but clonogenic radiation survival was superimposable with either the survival of AIIB2/JNKi or HDACi-treated cells (Figure 5D–5E, Supplementary Figure 9). These results indicate codependence between β1 integrin/JNK and HDACs on a mechanistic basis, explaining, at least partly, the radiosensitization elicited by simultaneous β1 integrin/JNK targeting.

### Simultaneous β1 integrin/JNK blocking enforces G2/M cell cycle phase arrest

**Figure 6:**
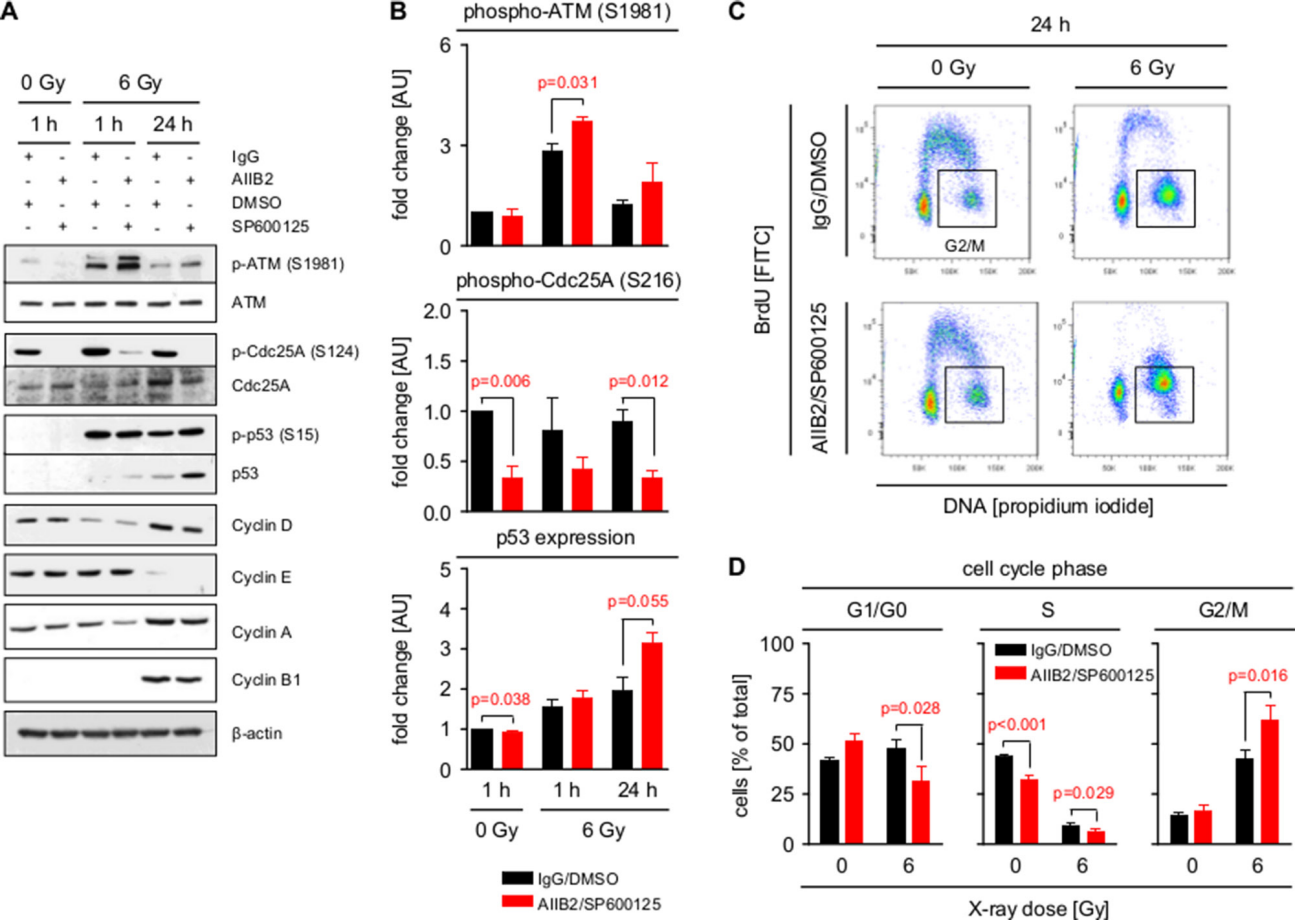
Inhibition of β1 integrin and JNK enhances G2/M cell cycle arrest (**A**) Western blot analysis of indicated proteins and phosphorylation sites from whole cell lysates of U343MG cells treated with AIIB2/SP600125 (EC10) or control IgG/DMSO without and with X-ray irradiation (6 Gy). Representative blots are shown. (**B**) Quantification of ATM (S1981) and Cdc25A (S216) phosphorylation and p53 expression shown in (A). Fold changes are calculated by normalization to β-actin and IgG/DMSO controls. Results are mean +/− SEM (*n* = 3, *t*-test). (**C**) Flow cytometric cell cycle analysis (BrdU, propidium iodide) of U343MG cells 24 h after treatment with AIIB2/SP600125 (EC10) or control IgG/DMSO without and with X-ray irradiation (6 Gy). Representative dot blots are shown. (**D**) Quantification of flow cytometric analysis from (C) showing distribution of treated GBM cells into G1/G0, S and G2/M phase of the cell cycle. Results are mean +/− SEM (*n* = 3, *t*-test).

**Figure 7:**
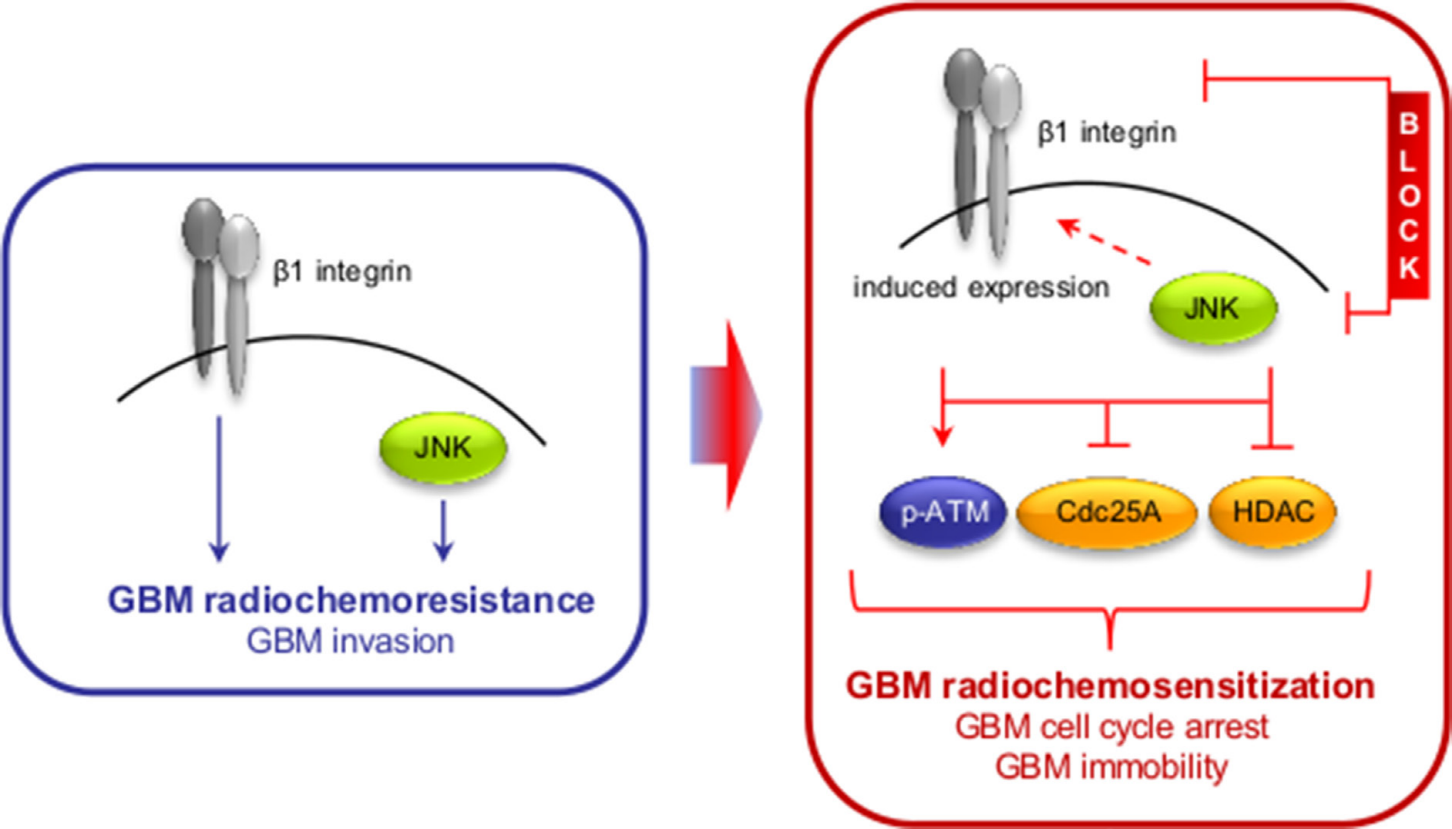
Scheme depicting the effect of the dual β1 integrin and JNK targeting approach on GBM radioresistance and invasion

Based on the function of HDAC for cell cycle arrest and progression [41], phosphorylation of a number of direct cell cycle regulators was also modified upon AIIB2/JNKi (Cdc25A S124, Chk2 T387, cyclin B1 S126, etc.) (Figure 4E–4F). This led us to analyze cell cycle signaling and distribution in AIIB2/JNKi-treated U343MG cells with and without irradiation. Interestingly, and in line with the increase in DNA DSB (Figure 5B–5C), we discovered that dual β1 integrin/JNK deactivation increases S1981 phosphorylation of the important DNA damage sensor ATM at 1 and 24 h post X-ray irradiation as compared to control-treated cells (Figure 6A–6B). Moreover, Cdc25A S124 phosphorylation strongly diminished downstream of ATM concomitant with stabilized radiation-dependent p53 expression upon β1 integrin/JNK targeting (Figure 6A–6B). Surprisingly, changes detected in cyclin phosphorylation (Figure 4F) did not lead to significant changes in cyclin expression between the treatment groups (Figure 6A). Intriguingly and despite unchanged cyclin expression, we found a significantly stronger G2/M phase arrest with parallel decreases in G1/G0 and S phase elicited by AIIB2/JNKi at 24 h after irradiation as compared with controls (Figure 6C–6D). Importantly, our data connect β1 integrin/JNK deactivation to molecular alterations leading to an enhancement of DSB and associated radiation-induced cell cycle modifications (Figure 7).

## DISCUSSION

Cancer therapy resistance arises as a result from different factors, mainly genetic alterations in cancer stem or bulk cells, changes in the tumor microenvironment and the plasticity to evade therapeutic intervention by initiation of prosurvival adaptation mechanisms. Understanding the treatment response of tumor cells on both the cellular and microenvironmental level may be favorable to counteract such adaptation mechanisms for developing novel therapeutic multi-targeting concepts. Here we show that (i) combined β1 integrin/JNK targeting induces radiosensitization superior to monotherapies and blocks GBM cell invasion, (ii) inhibition of β1 integrin itself or JNK induces β1 integrin expression putatively acting as part of an adaptation mechanism, (iii) simultaneous targeting of β1 integrin and JNK in combination with radiotherapy/Temozolomide significantly delays tumor growth and increases median survival in an orthotopic GBM mouse model, and (iv) mechanistically, β1 integrin/JNK co-inhibition impaires HDAC function leading to elevated levels of euchromatin and enhancement of DNA double strand breaks, thus orchestrating G2/M phase arrest for radiochemosensitization downstream of ATM.

While our study confirms reports that a single targeting of JNK is cytotoxic for GBM stem-like cells and PDC [19, 20], we show that GBM radioresistance cannot be overcome by monotherapy with a pharmacological JNK inhibitor or a β1 integrin function blocking antibody. This may be due to an increase in the expression of β1 integrin as part of an adaptation mechanism becoming apparent by a radioresistant phenotype in GBM [24, 30], HNSCC [12, 42] and breast carcinoma [43]. In line with this hypothesis, our data evidently show that the radioresistance *in vitro* can be overcome by a co-targeting of JNK and β1 integrin, which also delayed tumor growth and increased survival of orthotopic GBM mice *in vivo*. Previous reports describe a molecular connection between nuclear factor kappa-light-chain-enhancer of activated B-cells (NF-κB) and the regulation of β1 integrin expression [44, 45], which is known to engage in prosurvival crosstalk with JNK signaling to promote mesenchymal differentiation together with radioresistance of GBM [46]. In light of the failure of the αvβ3/β5 integrin-blocking peptidomimetic Cilengitide to improve patient outcome [29], it is a key finding of our study that also single targeting of β1 integrin using AIIB2 failed to radiosensitize GBM cells. As seen in Supplementary Figure 7B, this failure might arise additionally from diversely activated adaptation mechanisms upon β1 integrin inhibition, which are driven by p38 MAPK, PLCγ2 and RAS-GRF1 among others. Consequently, further unraveling of such adaptation mechanisms is highly likely to provide novel therapeutic possibilities for improving GBM therapy efficacy. Furthermore, although the blood brain barrier (BBB) is often compromised in GBM, the heterogeneous effect of the BBB on compound delivery must be considered for novel therapies [47].

Further, we demonstrate that the enhanced GBM cell radiosensitivity by simultaneous deactivation of β1 integrin and JNK is partially based on an impairment of HDAC function, associated DNA repair deficiency and cell cycle arrest. While a role in DNA repair has been reported for both JNK and β1 integrin [42], it was novel to understand that the targeting of these two molecules transferred into a delayed DSB repair kinetic in parallel to elevated chromatin acetylation as indicated by changes in H3 acetylation and trimethylated H3K4 upon HDAC dephosphorylation. In this regard, we and others have previously shown that the chromatin organization fundamentally impacts on both the formation and repair of radiation-induced DSB in the way that a more relaxed euchromatin is, on the one hand, more susceptible to irradiation-induced DNA damage and, on the other hand, allows easier access for repair enzymes than condensed heterochromatin [37–39]. The deficient repair faced by the GBM cells is also obvious in the prolonged G2/M phase arrest occurring upon the AIIB2/JNKi/radiotherapy regimen as a consequence of deregulated Cdc25A to p53 signaling commonly known as major regulator of cell cycling [48, 49]. Taking into consideration that β1 integrin/JNK co-inhibition induces chromatin to relax in parallel to DNA repair impairment, our data might form a reasonable basis how a higher number of DSB results in an elevated ATM phosphorylation and intensified G2/M cell cycle phase arrest for radiosensitization.

In addition to enhanced radiochemosensitivity, simultaneous β1 integrin/JNK targeting affected invasion, the second most critical GBM characteristic. At the first glance, this may not be surprising, as JNK has been linked to cellular motility by phosphorylation of paxillin, a focal adhesion adapter protein that interacts with β1 integrin [50]. Furthermore, integrin heterodimers containing the β1 integrin subunit are main receptors for collagen adhesion and are implicated in regulating cell migration and invasion [22]. Interestingly, the brain is an environment largely devoid of fibrillary ECM components and the deposition of type I collagen observed in GBM is therefore of major importance through two aspects [51]. First, type I collagen expression in extra- and intratumoral connective tissue may act as a pro-invasive guidance cue [52]. Second, type I collagen production by GBM cells may constitute an essential niche component for GBM stem-like cells, thus fostering main pro-survival adaptation mechanisms provoking radiochemoresistance [4, 36].

In conclusion, our data suggest that the dual inhibition of β1 integrin and JNK is effectively enhancing the eradication of both GBM stem-like and bulk cell populations when concomitantly administered to radiotherapy *in vitro* and radiochemotherapy *in vivo*. At the current stage, our described approach remains preclinical and normal tissue toxicities require further attention. Consequently, currently available therapeutic, humanized inhibitory anti-β1 integrin antibodies and JNK inhibitors, such as PGL5001 (for the treatment of inflammatory endometriosis;www.clinicaltrials.gov, identifier NCT01630252), need to be tested for safety and side effects. While future studies are warranted for in-depth untangling of integrin and JNK pathway interactions, the presented promising findings may be exploitable to target adaptation mechanisms expediting radiochemoresistance and invasion in GBM.

## MATERIALS AND METHODS

### Antibodies

Antibodies were purchased as indicated: ATM, Cdc25A, HP1α, H3, H3K4me3, phospho-cJun S63, cJun, phospho-p53 S15 (Cell Signaling Technology); β-actin (Sigma); phospho-JNK T183/Y185 for histology (Santa Cruz Biotechnology); β1 integrin, p53, BrdU (BD Biosciences); phospho-ATM S1981 (Rockland Immunochemicals); phospho-Cdc25A S126, β1 integrin for histology (Abcam); acetyl-H3, phospho-Histone H2A.X Ser139 (Merck Millipore); 53BP1 (Novus Biologicals).

### Cell culture

Human U343MG cells were obtained from the American Type Culture Collection (ATCC) and cultured in Basal Medium Eagle (Thermo Fisher Scientific), supplemented with 10% fetal calf serum, 10 mM HEPES, 2 mM L-glutamine, and 1% non-essential amino acids (all from GE Healthcare) at 37°C in a humidified atmosphere containing 5% CO_2_. The human DD-T4 cell line was grown in Dulbecco's Modified Eagle's Medium (plus GlutaMAX-I) supplemented with 10% fetal calf serum and 1% non-essential amino acids (all from GE Healthcare) at 37°C and 8.5% CO_2_. Human GBM stem-like cells GS-8 [53] were kindly provided by K. Lamszus (University Medical Center Hamburg-Eppendorf, Germany) via material transfer agreement and grown in stem cell medium: Neurobasal Medium (Thermo Fisher Scientific) supplemented with 2 mM L-glutamine (GE Healthcare), 32 U/ml Heparin (Merck Millipore), B27 supplement, 20 ng/ml human EGF and 20 ng/ml human FGFb (all from Thermo Fisher Scientific) at 37°C and 5% CO_2_. To generate GS-8_GFP/fLuc cells, lentiviral transduction of GS-8 cells was kindly performed as described [54] by M. Cartellieri (Faculty of Medicine Carl Gustav Carus, Technische Universität Dresden, Germany) using the lentiviral vector p6NST50 [55] containing the firefly luciferase gene.

### Preparation of patient-derived GBM cell cultures

The isolation of GBM cells from GBM surgical specimens was approved by the ethics committee of the Technische Universität Dresden, Germany (approval no. AZ152052013) and written consent. Samples were cut into < 1 mm^3^ fragments, washed with PBS (Thermo Fisher Scientific) and digested with Trypsin/EDTA (Sigma-Aldrich) for 15 min at 37°C. Digested fragments were resuspended in stem cell medium, filtered through a 40 μm cell strainer (Corning) and cells were seeded into tissue culture flasks. Cells were grown at 37°C and 5% CO_2_ and spheres were propagated by mechanical dissociation in stem cell medium.

### Sphere and colony formation assay

Measurement of sphere formation and clonogenicity [30, 56] was performed by plating single cells in type I collagen coated (1 μg/cm^2^, BD Biosciences) 6-well or 96-well cell culture dishes, respectively. After a 24 hours settling time, cells were treated with AIIB2 (10 μg/ml), SP600125 (2.5–200 μM, Santa Cruz Biotechnology), LBH589 (0.625–80 nM, Novartis AG, [39]) and combinations thereof for 24 hours and then washed. Cells were irradiated 1 h after addition of inhibitors with 2, 4 or 6 Gy single X-ray doses. Non-specific IgG isotype antibodies (10 μg/ml) or DMSO (equal amount as inhibitors, < 0.1 % v/v) (AppliChem) were used as control. After a cell line-dependent growth period, cell colonies were fixed with 80% EtOH, stained with Coomassie blue (Merck Millipore) and colonies containing > 50 cells were counted. After a 6 d growth period, spheres containing > 25 cells were microscopically counted [56].

### Radiation exposure

*In vitro* and *in vivo* X-ray irradiation was delivered at room temperature using single doses of 200 kV X-rays filtered with 0.5 mm Cu (Yxlon Y.TU 320; Yxlon, Hamburg, Germany). The dose-rate was approximately 1.3 Gy/min at 20 mA. The absorbed dose was measured using a Duplex dosimeter (PTW, Freiburg, Germany). Applied doses ranged from 0 to 6 Gy X-rays.

### 3D invasion assay

Multicellular GBM spheroids were generated by culturing 10^4^ cells per well in 96-well plates coated with 1% agarose. After 1 – 3 days, spheroids were embedded in 1 mg/ml 3D type I collagen [30] and treated as indicated. Images of spheroids were acquired using an Axioscope 2 microscope (Zeiss) immediately after plating to define the spheroid perimeter. Further images were acquired at 24 h, 48 h and 72 h. In these images, the distance of invaded cells emanating from the 0 h perimeter was measured on 8 different positions within the same spheroid using the Axiovision (LE) software (Zeiss). The mean distance in μm was calculated.

### Orthotopic GBM mouse model

For *in vivo* experiments, 10 to 12 week old male and female athymic nude mice (NMRI nu/nu) were obtained from the OncoRay animal facility (Faculty of Medicine Carl Gustav Carus, Technische Universität Dresden, Germany). Before, during and after treatment, animals were housed in the special pathogen-free facility of the OncoRay, Technische Universität Dresden, Germany. A constant temperature of 26°C, a 12 h light - 12 h dark electric cycle, water ad libitum and laboratory animal diet were provided for the animals. The animal facility and experiments were authorized by the Landesdirektion Dresden Germany, according to the German and Saxony animal welfare regulations (No. TVV 2014/28). Mice were anesthetized using 10 mg/kg body weight (bw) xylazine and 100 mg/kg bw ketamine. In total, 25000 GS-8_GFP/fLuc cells in 3 μl PBS were orthotopically transplanted into the right hemisphere of mice using a stereotactic device (Stoelting). Six days after intracranial injection, mice were randomly assigned to different treatment groups and treated for 3 cycles comprised of 10 mg/kg bw IgG or AIIB2, 15 mg/kg bw SP600125, 10 mg/kg bw TMZ and corresponding amount of DMSO. X-ray irradiation (4 Gy) of the whole brain commenced 3 h after treatment under anesthesia and protection of eyes and snout. Tumor growth was assessed based on luminescence and MRI and survival monitored. Animals with a declining condition were euthanized in accordance with the German and Saxony animal welfare guidelines and the whole brain was immediately excised for histological analysis.

### Luminescence and magnetic resonance imaging of orthotopic brain tumors

Tumor growth was examined twice weekly by bioluminescence imaging using the IVIS SPECTRUM (Perkin Elmer). For this analysis, mice were anesthetized using 2.0–2.5 % isoflurane in oxygen 10 min after i. p. injection of 150 ml/kg BW D-Luciferin (Thermo Fischer Scientific). Tumor growth was assessed by calculating the fold change of luminescence counts over time. Additionally, selected mice were subjected to MRI. MRI was performed 24 d after intracranial injection using a 1.0 tesla small animal scanner (nanoScan PET/MRI, Mediso Medical Imaging Systems, Budapest, Hungary) with the 35 mm whole body mouse coil. 2.0–2.5 % isoflurane in oxygen was used for anesthesia and mice were positioned in a whole body mouse bed with an integrated warming system. Bed temperature (36°C) and breathing frequency were monitored during the whole imaging procedure. A 3D gradient echo spoiled T1 weighted sequence was performed 10 min after i. p. injection of 5 ml/kg BW Omniscan^®^ (GE Healthcare) with a field of view covering the head of the mouse. The sequence parameters are: repetition time (TR) = 15 ms, echo time (TE) = 3.1 ms, flip angle = 25°, field of view = 60 mm, slice thickness = 0.23 mm and number of slices = 90. Data was analyzed using InterviewFusion^TM^ (Mediso Medical Imaging Systems).

### Histology

For immunofluorescence analysis of brain sections brains of mice were fixed overnight in 4% formalin and embedded in paraffin. Paraffin sections were deparaffinized in xylene and rehydrated. Antigen retrieval was performed in 10 mM citric acid, pH 6.0, at 98°C for 15 min and sections were stained with hematoxylin and eosin or antibodies against β1 integrin and phospho-JNK T183/Y185. For tyramide based immunofluorescence detection, the TSA-kit (Thermo Fisher Scientific) was used according to the manufacturer's instructions. Sections were mounted using ProLong Gold Antifade Mountant with DAPI (Thermo Fisher Scientific) for nuclear counterstaining. Images were acquired using an AxioImager M1 microscope or LSM510meta (Zeiss).

### Phosphoproteome analysis

U343MG cells were cultured on type I collagen for 24 h and treated either with AIIB2/DMSO (10 μg/ml), IgG/SP600125 (EC10), AIIB2/SP600125 (EC10) or control IgG/DMSO. Cells were harvested after 1 h and whole cell lysates prepared. The Phospho Explorer Antibody Microarray was conducted by Full Moon BioSystems Inc., as previously published [12]. Additional details are described in the Supplementary Methods.

### Cell cycle analysis

GBM cells were cultured on type I collagen for 24 h and treated as indicated. Before harvesting, cells were incubated with 10 mM BrdU (BD Biosciences) for 10 min and then prepared for cell cycle measurement. Cells were harvested using 1x trypsin/EDTA and fixed in icecold 80% ethanol until use. Cells were further prepared for analysis by incubation with 0.01 % RNase A, 2 N HCl (all Sigma-Aldrich) and 0.5% Triton-X-100/PBS (Carl Roth GmbH). Subsequently, mouse anti-BrdU antibodies and propidium iodide (Sigma-Aldrich) was added for the detection of incorporated BrdU and total DNA content. Cell cycle distribution was determined using an FACS Calibur (BD Biosciences) and analyzed using the FlowJo Software (Flowjo LLC).

### DSB analysis

For the detection of DNA DSB, 4 × 10^5^ cells were plated in T25 tissue culture flasks coated with type I collagen for 24 h and treated as indicated. Cells were isolated using 1 × trypsin/EDTA cells were and fixed with 3% formaldehyde (Merck KGaA) and permeabilized with 0.25% Triton-X-100/PBS. Staining was accomplished with specific antibodies for 53BP1. Foci were counted by two independent investigators using an Axioscope1 plus fluorescence microscope (Zeiss). Immunofluorescence images were acquired using LSM 510 meta confocal microscope (Zeiss).

### Statistical analysis

Means +/− SEM of at least three independent experiments were calculated with reference to controls defined in total numbers or 1.0. For statistical analysis of clonogenic survival, invasion, densitometry, cell cycle and DSB, two-sided Student's *t*-test was performed using Excel (Microsoft). The *p*-values for radiation-dependent sphere and colony formation are shown in Supplementary Table 1. For statistical analysis of animal experiments, an analysis of variance (ANOVA) and two-sided log rank test were performed with Prism (GraphPad Software). A *P* value of less than 0.05 was considered statistically significant.

## SUPPLEMENTARY MATERIALS FIGURES AND TABLES





## Abbreviations

GBM: Glioblastoma
JNK: c-Jun N-terminal kinase
RTK: receptor tyrosine kinase
TMZ: Temozolomide
ECM: extracellular matrix
MMP: matrix metalloproteinase
MRI: magnetic resonance imaging
RCT: radiochemotherapy
