# Supplementary material for: Adhesion- and stress-related adaptation of glioma radiochemoresistance is circumvented by β1 integrin/JNK co-targeting
Source: Oncotarget. 2017 Apr 27;8(30):49224–37. doi: 10.18632/oncotarget.17480 (PMC5564763; doi:10.18632/oncotarget.17480)
Supplement: Supplementary file 1 [file oncotarget-08-49224-s001.pdf]

Adhesion- and stress-related adaptation of glioma radiochemoresistance is circumvented by β1 integrin/JNK co-targeting

Supplementary Materials

SUPPLEMENTARY METHODS

Total protein extracts and western blotting

Cells were lysed with modified RIPA buffer consisting of 50 mM Tris-HCl (pH 7.4), 1% Nonidet-P40, 0.25% sodium deoxycholate, 150 mM NaCl, 1 mM EDTA, 1 mM $NaVO_4$, 2 mM NaF (all Sigma-Aldrich), Complete protease inhibitor cocktail (Roche). Total protein amount was measured by BCA assay (Thermo Fisher Scientific). After SDS–PAGE and transfer of proteins onto nitrocellulose membranes (GE Healthcare), probing of specific proteins was accomplished using indicated primary antibodies and horseradish peroxidase-conjugated donkey anti-rabbit and sheep anti-mouse antibodies (GE Healthcare). Enhanced chemiluminescent reagent (Amersham) was used for detection of proteins on X-ray films (GE Healthcare).

Phosphoproteome analysis

Proteins with phosphorylation site changes of at least 30% reduction or 50% enhancement are shown in the clustered image map created using CIMminer (https://discover.nci.nih.gov/cimminer/). Proteins falling within the defined threshold upon AIIB2/SP600125 treatment were classified for functionality according to their membership in known cancer signaling pathways (path:hsa05200; KEGG pathways in cancer). Corresponding pathway gene lists were compiled using ConsensusPathDB [1] and a summary of considered cancer signaling pathways has been published [2]. Fisher's exact test was used to quantify the enrichment of genes with increased or decreased phosphorylation in each signaling pathway. The molecular interaction network of β1 integrin, JNK and cell cycle regulatory proteins was created using Cytoscape (Cytoscape Consortium, [3]).

Transcriptome analysis

Analysis of mRNA expression of integrin subunits, ECM ligands and JNK isoforms in GBM and normal brain was performed on indicated studies using the Oncomine database [4–6].

REFERENCES

1. Kamburov A, Pentchev K, Galicka H, Wierling C, Lehrach H, Herwig R. ConsensusPathDB: toward a more complete picture of cell biology. Nucleic Acids Res. 2011; 39:D712–7. doi: 10.1093/nar/gkq1156.
2. Seifert M, Abou-El-Ardat K, Friedrich B, Klink B, Deutsch A. Autoregressive higher-order hidden Markov models: exploiting local chromosomal dependencies in the analysis of tumor expression profiles. PLoS One. 2014; 9:e100295. doi: 10.1371/journal.pone.0100295.
3. Shannon P, Markiel A, Ozier O, Baliga NS, Wang JT, Ramage D, Amin N, Schwikowski B, Ideker T. Cytoscape: a software environment for integrated models of biomolecular interaction networks. Genome Res. 2003; 13:2498–504. doi: 10.1101/gr.1239303.
4. Rhodes DR, Yu J, Shanker K, Deshpande N, Varambally R, Ghosh D, Barrette T, Pandey A, Chinnaiyan AM. ONCOMINE: a cancer microarray database and integrated data-mining platform. Neoplasia. 6:1–6.
5. Sun L, Hui AM, Su Q, Vortmeyer A, Kotliarov Y, Pastorino S, Passaniti A, Menon J, Walling J, Bailey R, Rosenblum M, Mikkelsen T, Fine HA. Neuronal and glioma-derived stem cell factor induces angiogenesis within the brain. Cancer Cell. 2006; 9:287–300. doi: 10.1016/j.ccr.2006.03.003.
6. Bredel M, Bredel C, Juric D, Harsh GR, Vogel H, Recht LD, Sikic BI. High-Resolution Genome-Wide Mapping of Genetic Alterations in Human Glial Brain Tumors. Cancer Res. 2005; 65:4088–96. doi: 10.1158/0008-5472.CAN-04-4229.
7. Yan H, Zhang B, Li S, Zhao Q. A formal model for analyzing drug combination effects and its application in TNF-α-induced NFκB pathway. BMC Syst Biol. 2010; 4:50. doi: 10.1186/1752-0509-4-50.

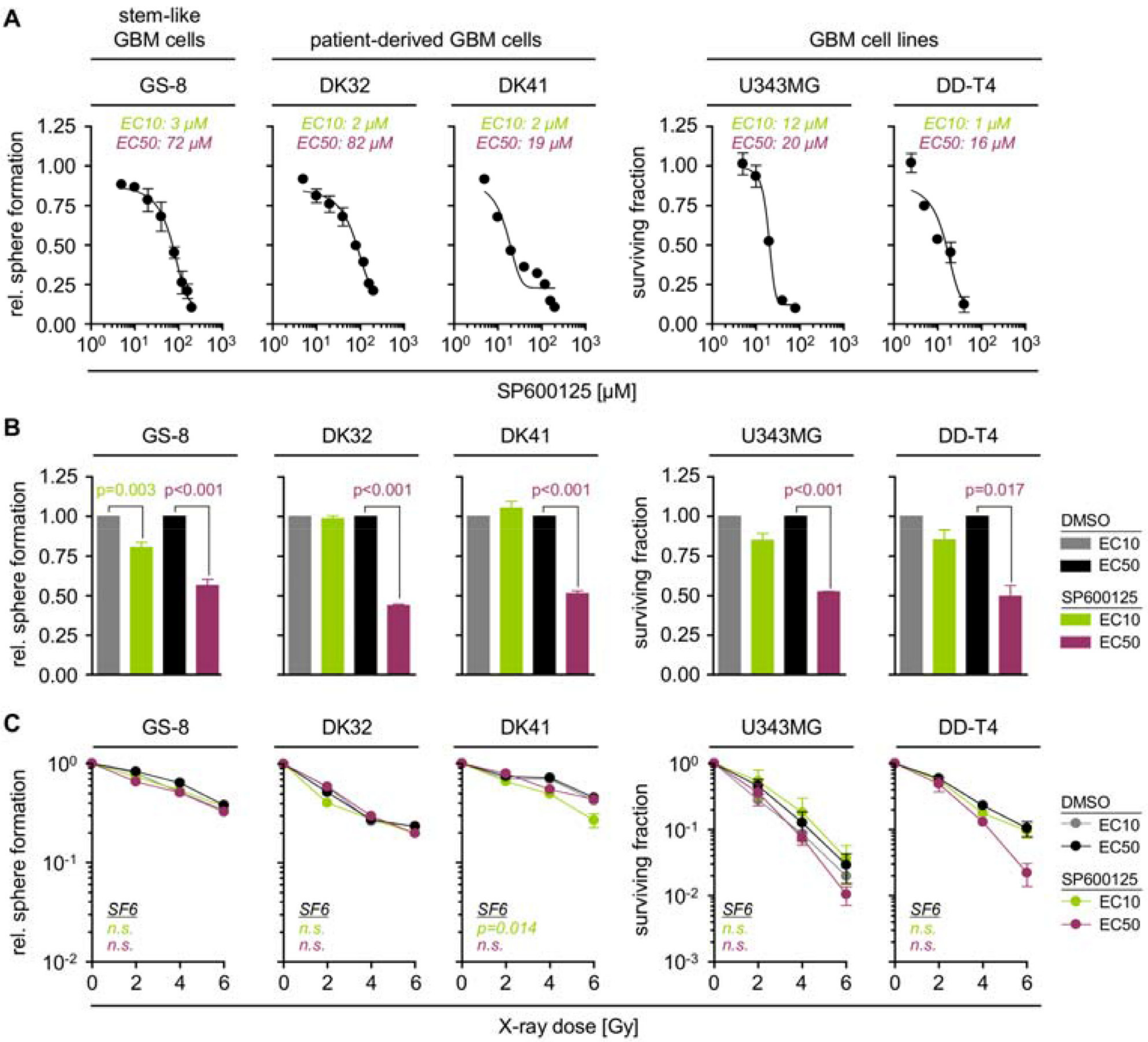

Supplementary Figure 1: JNK inhibition in GBM stem-like cells, patient-derived GBM cell cultures and GBM cell lines. (**A**) Relative sphere formation and basal surviving fraction of indicated GBM cells upon treatment with the indicated doses of SP600125 compared to control (DMSO). EC10 and EC50 values are shown. Results are mean +/− SEM (*n* = 3). (**B**) Relative sphere formation and basal surviving fraction of indicated GBM cells upon treatment with SP600125 (EC10, EC50) or control (DMSO). (**C**) Relative sphere formation and clonogenic survival upon treatment with SP600125 or control and X-ray irradiation (2, 4, 6 Gy). (B–C) Results are mean +/− SEM (*n* = 3–4, *t*-test).

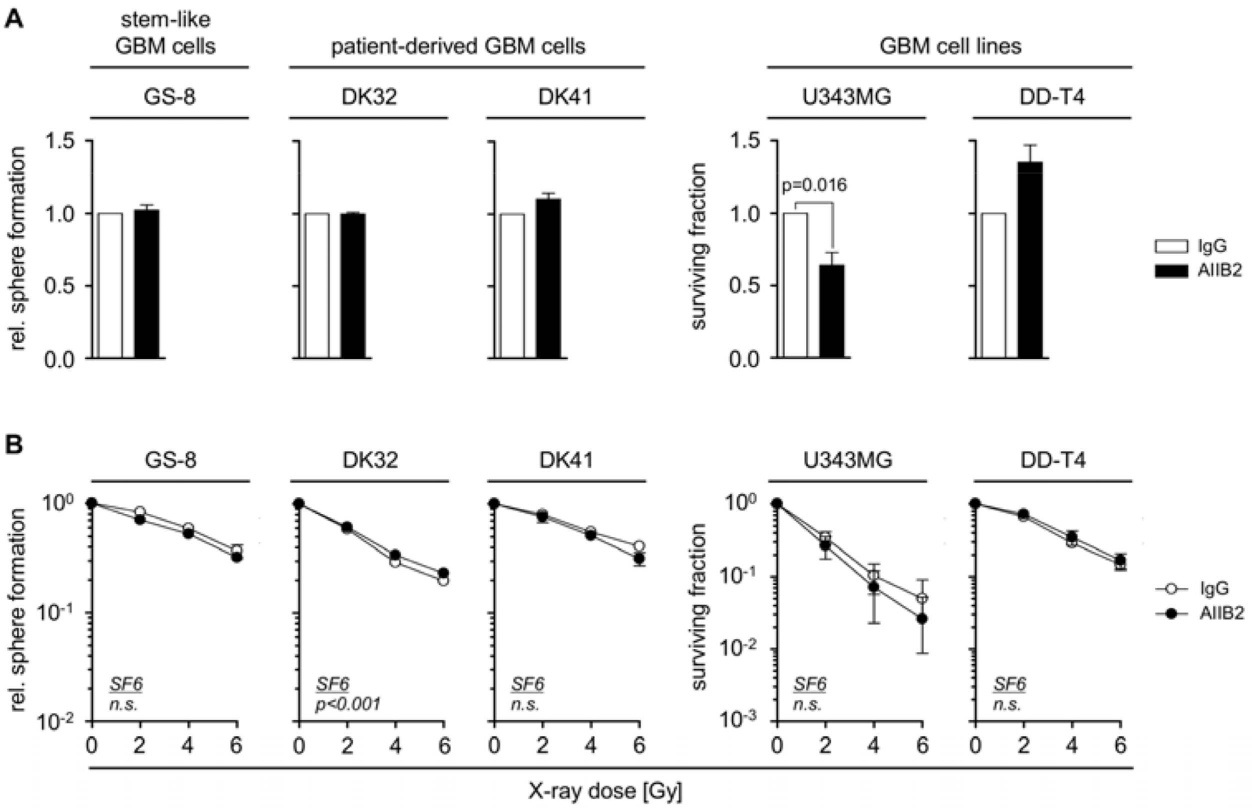

Supplementary Figure 2: β1 integrin inhibition in GBM cell cultures. (**A**) Relative sphere formation and basal surviving fraction of indicated GBM cells upon treatment with AIIB2 or control IgG (both 10 µg/ml). (**B**) Relative sphere formation and clonogenic survival upon treatment with AIIB2 or control and X-ray irradiation (2, 4, 6 Gy). (A, B) Results are mean +/− SEM (*n* = 3–4, *t*-test).

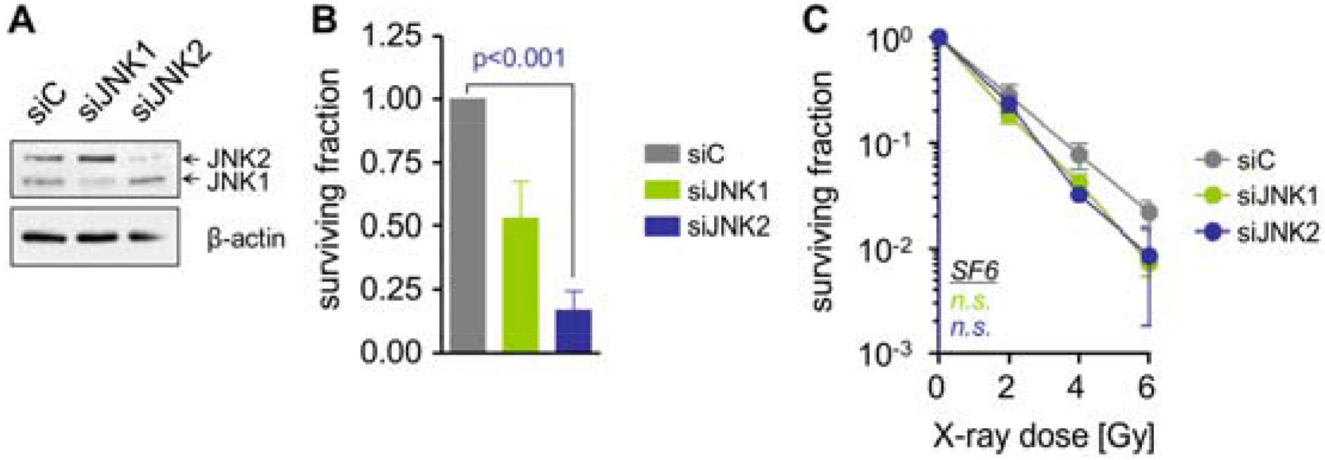

Supplementary Figure 3: siRNA-mediated JNK targeting. (**A**) Analysis of JNK1, JNK2 and β-actin expression in whole U343MG cell lysates after siRNA-mediated knockdown of JNK1 or JNK2 or control siRNA. (**B**) Basal surviving fraction and (**C**) clonogenic survival upon X-ray irradiation (2, 4, 6 Gy) of U343MG cells upon siRNA-mediated depletion of JNK1 or JNK2 relative to control siRNA. (B, C) Results are mean +/− SEM (*n* = 3, *t*-test).

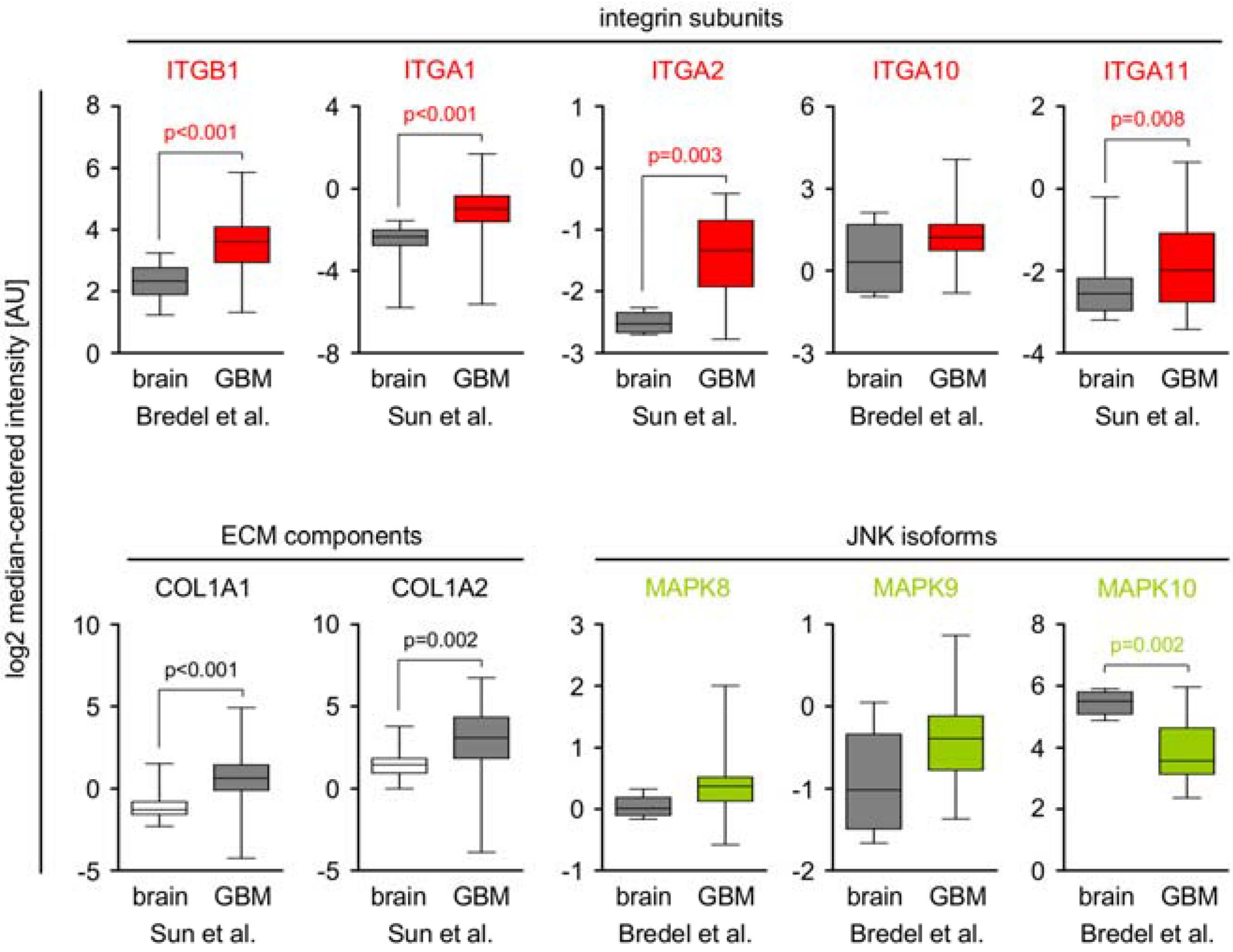

Supplementary Figure 4: β1 integrins, associated a integrin subunits and their ECM ligands are upregulated in GBM. Oncomine-based comparative analysis of mRNA levels of normal brain and GBM showing β1 integrin and associated α integrin subunits, specific ECM ligands for indicated integrins and JNK isoforms (*t*-test) [4–6].

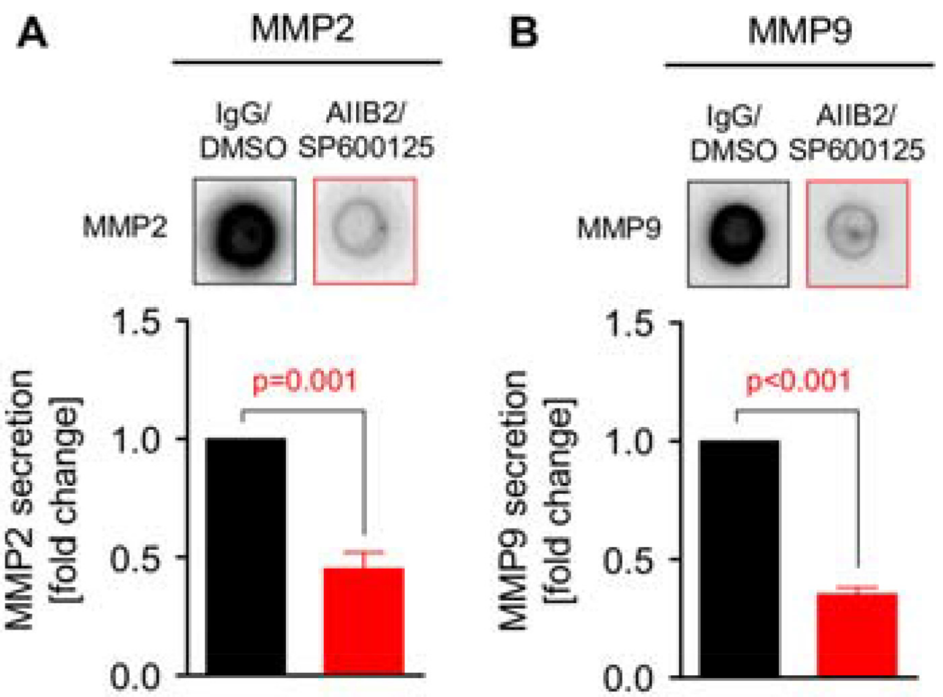

Supplementary Figure 5: Combined β1 integrin/JNK targeting reduces secretion of MMP2 and MMP9 in GBM. Representative images depict dot blot analysis of (**A**) MMP2 and (**B**) MMP9 protein levels in supernatants of U343MG cells treated with AIIB2/SP600125 (EC10) for 1 h (IgG/DMSO as control). (A, B) Graphs show densitometric quantification thereof. Results are mean +/− SEM (*n* =3, *t*-test).

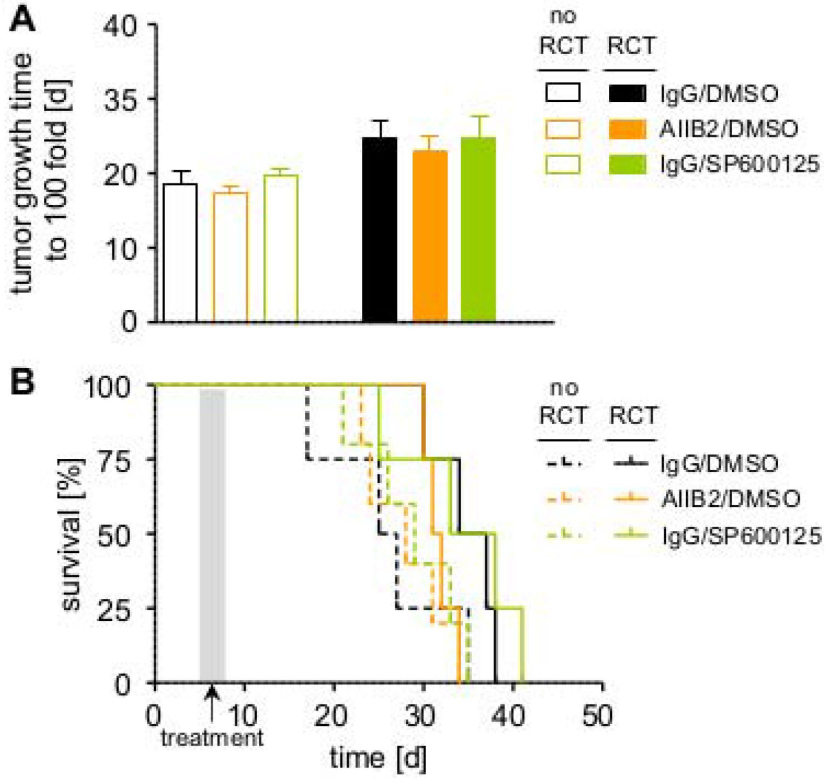

Supplementary Figure 6: Single targeting of β1 integrin and JNK in combination with radiochemotherapy. (**A**) GS-8_GFP/fLuc tumor growth time to 100 fold radiance after β1 integrin or JNK inhibition without or in combination with radiochemotherapy (RCT) as indicated. Data are mean +/− SEM. (**B**) Survival of GS-8_GFP/fLuc mice treated as indicated. Kaplan Meier analysis includes 6 mice IgG/DMSO, 7 mice AIIB2/DMSO, 9 mice IgG/SP600125, 6 mice IgG/DMSO+RCT, 6 mice AIIB2/DMSO+RCT, 4 mice IgG/SP600125+RCT (two-sided log rank test: not significant).

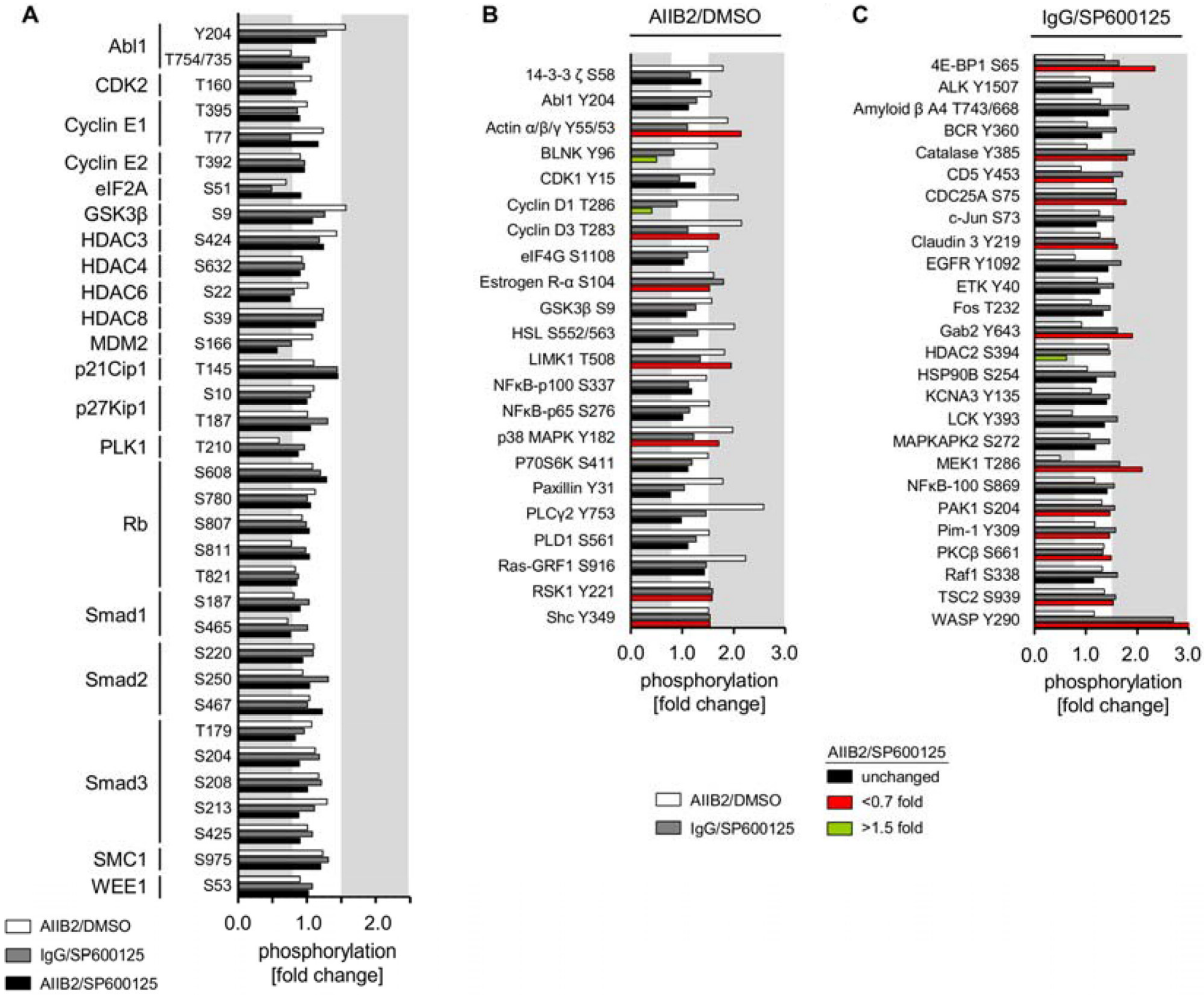

Supplementary Figure 7: Dual targeting of β1 integrin and JNK and cell cycle regulatory networks. Fold change in phosphorylation of (**A**) cell cycle associated proteins from phosphoproteome analysis of U343MG (Figure 4), (**B**) phosphosites with increased phosphorylation upon AIIB2/DMSO treatment and (**C**) phosphosites with increased phosphorylation upon IgG/SP600125 treatment. (A–C) Range (30% decrease, 50% increase) of fold change in phosphorylation is marked by grey area.

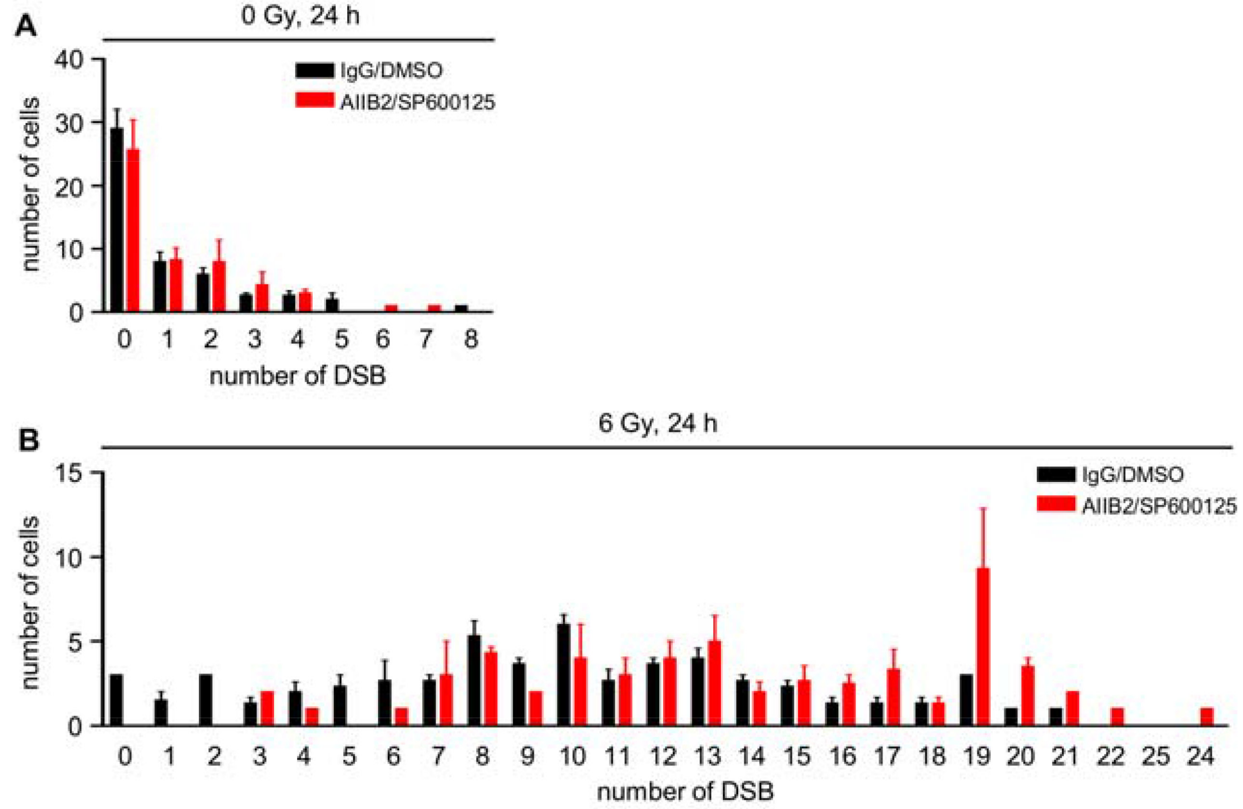

Supplementary Figure 8: β1 integrin and JNK deactivation impairs DSB repair. (**A–B**) Quantification of the number of cells with indicated number of residual DSB 24 h after treatment with (A) IgG/DMSO or (B) AIIB2/SP600125 (EC10) with X-ray irradiation (6 Gy). Results are mean +/− SEM (*n* = 3).

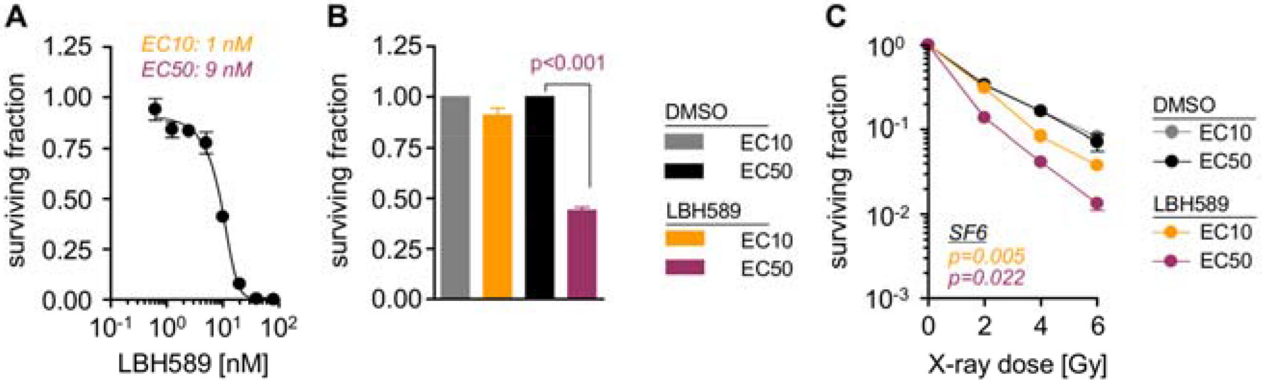

Supplementary Figure 9: LBH589 cytotoxicity in GBM cells. (A) Cytotoxicity of LBH589 in U343MG cells (DMSO served as control). EC10 and EC50 values are indicated. (B) Basal surviving fraction of U343MG cells upon treatment with the HDAC inhibitor LBH589 (EC10, EC50) or control (DMSO). (C) Clonogenic radiation survival of U343MG cells treated as described in (B). (A–C) Results are mean +/− SEM (*n* = 3, *t*-test).

Supplementary Table 1: *p*-values for clonogenic radiation survival of cell lines in indicated figures and figure supplements.

Treatment (vs. control)	Cell line	SF2	SF4	SF6
**Figure 1c**				
AIIB2/SP600125 EC10	GS-8	0.056	0.018	0.002
	DK32	0.000	0.000	0.000
	DK41	0.010	0.006	0.001
	U343MG	0.716	0.115	0.041
	DD-T4	0.436	0.131	0.000
AIIB2/SP600125 EC50	GS-8	0.043	0.026	0.046
	DK32	0.096	0.000	0.000
	DK41	0.131	0.155	0.000
	U343MG	0.407	0.037	0.012
	DD-T4	0.646	0.016	0.054
**Figure 6e**				
AIIB2/SP600125	U343MG	0.050	0.000	0.015
LBH589	U343MG	0.001	0.005	0.022
AIIB2/SP600125/LBH589	U343MG	0.000	0.000	0.043
**Supplementary Figure 1C**				
SP600125 EC10	GS-8	0.211	0.576	0.491
	DK32	0.007	0.128	0.101
	DK41	0.565	0.017	0.014
	U343MG	0.419	0.574	0.519
	DD-T4	0.455	0.142	0.809
SP600125 EC50	GS-8	0.065	0.074	0.131
	DK32	0.016	0.375	0.100
	DK41	0.491	0.024	0.738
	U343MG	0.603	0.479	0.307
	DD-T4	0.471	0.034	0.082
**Supplementary Figure 2B**				
AIIB2	GS-8	0.031	0.299	0.457
	DK32	0.084	0.008	0.000
	DK41	0.691	0.544	0.151
	U343MG	0.517	0.656	0.656
	DD-T4	0.577	0.542	0.668
**Supplementary Figure 3C**				
siJNK1	U343MG	0.294	0.194	0.077
siJNK2	U343MG	0.373	0.431	0.871
**Supplementary Figure 8C**				
LBH589 EC10	U343MG	0.404	0.011	0.005
LBH589 EC50	U343MG	0.001	0.005	0.022

Supplementary Table 2: Calculation of the combination effect of β1 integrin and JNK inhibition as published [7] using the following formula: S = r(a, b) – r(a0, b) x r(a, b0).

		GS-8	DK32	DK41	U343MG	DD-T4
**AIIB2 r(a, b0)**	**0 Gy**	0.0300	0.0653	0.0226	0.0974	0.0926
	**2 Gy**	0.0212	0.0398	0.0167	0.0332	0.0672
	**4 Gy**	0.0157	0.0222	0.0113	0.0113	0.0323
	**6 Gy**	0.0096	0.0151	0.0069	0.0044	0.0157
**JNKi (EC50) r(a0, b)**	0 Gy	0.0153	0.0289	0.0095	0.1893	0.0199
	**2 Gy**	0.0099	0.0171	0.0076	0.0469	0.0092
	**4 Gy**	0.0077	0.0086	0.0051	0.0101	0.0025
	**6 Gy**	0.0049	0.0057	0.0041	0.0021	0.0004
**AIIB2/JNKi (EC50) r(a, b)**	**0 Gy**	0.0131	0.0340	0.0141	0.0444	0.0140
	**2 Gy**	0.0071	0.0159	0.0080	0.0126	0.0094
	**4 Gy**	0.0038	0.0054	0.0061	0.0016	0.0019
	**6 Gy**	0.0018	0.0042	0.0022	0.0001	0.0003
**S**	**0 Gy**	0.0126	0.0321	0.0138	0.0260	0.0122
	**2 Gy**	0.0068	0.0153	0.0078	0.0111	0.0088
	**4 Gy**	0.0037	0.0052	0.0061	0.0015	0.0019
	**6 Gy**	0.0017	0.0041	0.0021	0.0001	0.0003

(Synergistic effect: S < 0, Additive effect: S = 0, Antagonistic effect: S > 0).

Supplementary Table 3: Signal ratio of phosphorylated protein to non-phosphorylated protein from phosphoproteome array data of U343MG cells 1 h after treatment with AIIB2/SP600125 (EC10) or IgG/DMSO. See Supplementray_Table_3

Supplementary Table 4: List of altered phospho-sites (30% decreased or 50% increased phosphorylation) from phosphoproteome analysis of U343-MG cells 1 h after indicated treatment in comparison to the control IgG/DMSO treatment as shown in Figure 4A. See Supplementray_Table_4
