# Supplementary material for: Adhesion- and stress-related adaptation of glioma radiochemoresistance is circumvented by β1 integrin/JNK co-targeting
Source: Oncotarget. 2017 Apr 27;8(30):49224–37. doi: 10.18632/oncotarget.17480 (PMC5564763; doi:10.18632/oncotarget.17480)
Supplement: Supplementary file 2 [file oncotarget-08-49224-s002.docx]

**Supplementary table 3**

Signal ratio of phosphorylated protein to non-phosphorylated protein from phosphoproteome array data of U343MG cells 1 h after treatment with AIIB2/SP600125 (EC10) or IgG/DMSO.

| **phosphorylation sites** | **IgG/DMSO** | **AIIB2/DMSO** | **IgG/SP600125** | **AIIB2/SP600125** |
| --- | --- | --- | --- | --- |
| 14-3-3 beta/zeta (Phospho-Ser184/186) | 0.61 | 0.63 | 0.70 | 0.88 |
| 14-3-3 theta/tau (Phospho-Ser232) | 1.79 | 1.65 | 1.69 | 1.96 |
| 14-3-3 zeta (Phospho-Ser58) | 0.92 | 1.65 | 1.08 | 1.26 |
| 14-3-3 zeta/delta (Phospho-Thr232) | 0.22 | 0.21 | 0.17 | 0.09 |
| 4E-BP1 (Phospho-Thr36) | 1.21 | 0.99 | 0.92 | 0.97 |
| 4E-BP1 (Phospho-Thr45) | 0.91 | 0.73 | 0.83 | 0.81 |
| 4E-BP1 (Phospho-Ser65) | 0.04 | 0.05 | 0.06 | 0.08 |
| 4E-BP1 (Phospho-Thr70) | 0.81 | 0.73 | 0.72 | 0.62 |
| 6-phosphofructo-2-kinase/fructose-2,6-biphosphatase 2 (PFKFB2) (Phospho-Ser483) | 0.50 | 0.47 | 0.53 | 0.56 |
| Abl1 (Phospho-Tyr204) | 0.02 | 0.03 | 0.02 | 0.02 |
| Abl1 (Phospho-Thr754/735) | 1.23 | 0.95 | 1.27 | 1.15 |
| ACC1 (Phospho-Ser79) | 0.65 | 0.60 | 0.84 | 0.53 |
| ACC1 (Phospho-Ser80) | 0.30 | 0.30 | 0.21 | 0.20 |
| Actin Pan (a/b/g) (Phospho-Tyr55/53) | 0.24 | 0.46 | 0.27 | 0.52 |
| ADD1 (Phospho-Ser726) | 1.53 | 1.69 | 1.75 | 1.29 |
| AFX/FOXO4 (Phospho-Ser197) | 0.58 | 0.77 | 0.61 | 0.64 |
| AKT1 (Phospho-Ser124) | 0.56 | 0.43 | 0.37 | 0.43 |
| AKT1 (Phospho-Ser246) | 1.50 | 1.94 | 1.44 | 1.67 |
| AKT1 (Phospho-Thr308) | 0.57 | 0.55 | 0.59 | 0.56 |
| AKT1 (Phospho-Tyr326) | 0.67 | 0.61 | 0.57 | 0.55 |
| AKT1 (Phospho-Thr450) | 0.85 | 0.82 | 0.71 | 0.69 |
| AKT1 (Phospho-Ser473) | 0.66 | 0.64 | 0.74 | 0.69 |
| AKT1 (Phospho-Tyr474) | 1.63 | 1.82 | 1.59 | 1.74 |
| AKT1 (Phospho-Thr72) | 0.77 | 0.80 | 0.69 | 0.81 |
| AKT1S1 (Phospho-Thr246) | 0.22 | 0.22 | 0.19 | 0.13 |
| AKT2 (Phospho-Ser474) | 1.62 | 1.64 | 1.78 | 1.92 |
| ALK (Phospho-Tyr1507) | 2.15 | 2.33 | 3.31 | 2.42 |
| ALK (Phospho-Tyr1604) | 1.00 | 1.21 | 0.86 | 0.59 |
| AMPK beta1 (Phospho-Ser182) | 0.57 | 0.66 | 0.47 | 0.33 |
| AMPK1 (Phospho-Thr172) | 0.87 | 0.93 | 0.83 | 0.92 |
| AMPK1/AMPK2 (Phospho-Ser485/491) | 1.06 | 1.24 | 0.91 | 1.10 |
| Amyloid beta A4 (Phospho-Thr743/668) | 0.31 | 0.39 | 0.56 | 0.44 |
| Androgen Receptor (Phospho-Ser213) | 0.99 | 1.02 | 1.06 | 0.97 |
| Androgen Receptor (Phospho-Ser650) | 0.49 | 0.46 | 0.48 | 0.49 |
| A-RAF (Phospho-Tyr301/302) | 0.62 | 0.62 | 0.64 | 0.66 |
| Arrestin-1 (Phospho-Ser412) | 0.90 | 0.93 | 0.86 | 0.78 |
| ASK1 (Phospho-Ser83) | 1.19 | 1.41 | 1.13 | 1.11 |
| ASK1 (Phospho-Ser966) | 0.52 | 0.34 | 0.35 | 0.29 |
| ATF1 (Phospho-Ser63) | 0.38 | 0.30 | 0.43 | 0.50 |
| ATF2 (Phospho-Ser112/94) | 1.11 | 1.15 | 1.06 | 1.14 |
| ATF2 (Phospho-Ser62/44) | 0.08 | 0.12 | 0.11 | 0.10 |
| ATF2 (Phospho-Thr69/51) | 0.90 | 0.99 | 0.87 | 0.98 |
| ATF2 (Phospho-Thr71/53) | 2.63 | 1.96 | 1.77 | 1.98 |
| ATF2 (Phospho-Thr73/55) | 1.23 | 1.10 | 1.17 | 1.14 |
| ATF4 (Phospho-Ser245) | 0.95 | 0.93 | 1.11 | 1.02 |
| ATP1A1/Na+K+ ATPase1 (Phospho-Ser23) | 1.66 | 1.31 | 1.75 | 1.37 |
| ATPase (Phospho-Ser16) | 0.65 | 0.53 | 0.66 | 0.70 |
| ATP-Citrate Lyase (Phospho-Ser454) | 0.56 | 0.56 | 0.65 | 0.66 |
| ATRIP (Phospho-Ser68/72) | 0.03 | 0.03 | 0.03 | 0.02 |
| AurA (Phospho-Thr288) | 0.98 | 0.83 | 0.86 | 0.80 |
| AurA (Phospho-Ser342) | 0.74 | 0.69 | 0.82 | 0.65 |
| AurB (Phospho-Tyr12) | 1.03 | 0.86 | 1.07 | 0.97 |
| AurB (Phospho-Thr232) | 0.84 | 0.78 | 0.89 | 0.84 |
| BAD (Phospho-Ser112) | 0.73 | 0.88 | 0.84 | 0.83 |
| BAD (Phospho-Ser134) | 0.77 | 0.68 | 0.59 | 0.66 |
| BAD (Phospho-Ser136) | 1.05 | 1.17 | 0.95 | 1.03 |
| BAD (Phospho-Ser155) | 0.94 | 1.16 | 1.03 | 1.00 |
| BAD (Phospho-Ser91/128) | 1.04 | 0.77 | 0.92 | 0.86 |
| BAX (Phospho-Thr167) | 1.40 | 1.67 | 1.22 | 1.20 |
| BCL-2 (Phospho-Thr56) | 0.97 | 1.11 | 1.10 | 1.09 |
| BCL-2 (Phospho-Thr69) | 1.34 | 1.58 | 1.36 | 1.40 |
| BCL-2 (Phospho-Ser70) | 1.56 | 2.22 | 1.62 | 1.59 |
| BCL-2 (Phospho-Ser87) | 1.45 | 1.68 | 1.44 | 1.33 |
| BCL-XL (Phospho-Ser62) | 0.09 | 0.09 | 0.11 | 0.12 |
| BCL-XL (Phospho-Thr47) | 0.03 | 0.03 | 0.02 | 0.03 |
| BCR (Phospho-Tyr177) | 1.02 | 1.40 | 1.35 | 1.24 |
| BCR (Phospho-Tyr360) | 1.63 | 1.68 | 2.59 | 2.14 |
| BID (Phospho-Ser78) | 0.84 | 0.66 | 0.79 | 0.75 |
| BIM (Phospho-Ser69/65) | 1.10 | 1.18 | 1.14 | 1.03 |
| BLNK (Phospho-Tyr84) | 0.55 | 0.74 | 0.49 | 0.31 |
| BLNK (Phospho-Tyr96) | 0.47 | 0.78 | 0.39 | 0.23 |
| B-RAF (Phospho-Ser446) | 1.07 | 1.02 | 0.96 | 0.94 |
| B-RAF (Phospho-Thr598) | 0.10 | 0.09 | 0.08 | 0.11 |
| B-RAF (Phospho-Ser601) | 0.87 | 0.96 | 0.85 | 0.81 |
| BRCA1 (Phospho-Ser1457) | 0.61 | 0.73 | 0.71 | 0.86 |
| BRCA1 (Phospho-Ser1524) | 0.51 | 0.41 | 0.50 | 0.49 |
| BTK (Phospho-Tyr223) | 1.38 | 1.01 | 0.93 | 1.32 |
| c-Abl (Phospho-Tyr412) | 0.54 | 0.52 | 0.56 | 0.54 |
| Calmodulin (Phospho-Thr79/Ser81) | 0.60 | 0.65 | 0.56 | 0.62 |
| Calsenilin/KCNIP3 (Phospho-Ser63) | 1.20 | 1.11 | 1.73 | 1.29 |
| CaMK1-alpha (Phospho-Thr177) | 0.63 | 0.57 | 0.60 | 0.65 |
| CaMK2A (Phospho-Thr286) | 0.61 | 0.58 | 0.66 | 0.69 |
| CaMK2-beta/gamma/delta (Phospho-Thr287) | 0.10 | 0.10 | 0.10 | 0.09 |
| CaMK4 (Phospho-Thr196/200) | 1.08 | 0.79 | 1.10 | 1.06 |
| Caspase 1 (Phospho-Ser376) | 1.70 | 1.78 | 1.80 | 1.65 |
| Caspase 2 (Phospho-Ser157) | 1.11 | 1.09 | 1.03 | 0.91 |
| Caspase 3 (Phospho-Ser150) | 1.38 | 1.53 | 1.30 | 1.06 |
| Caspase 6 (Phospho-Ser257) | 0.46 | 0.51 | 0.46 | 0.44 |
| Caspase 8 (Phospho-Ser347) | 0.70 | 0.56 | 0.64 | 0.70 |
| Caspase 9 (Phospho-Thr125) | 0.66 | 0.57 | 0.69 | 0.58 |
| Caspase 9 (Phospho-Ser144) | 1.04 | 0.99 | 1.18 | 1.08 |
| Caspase 9 (Phospho-Tyr153) | 0.58 | 0.30 | 0.16 | 0.53 |
| Caspase 9 (Phospho-Ser196) | 0.17 | 0.12 | 0.07 | 0.08 |
| Catalase (Phospho-Tyr385) | 0.27 | 0.28 | 0.53 | 0.49 |
| Catenin beta (Phospho-Ser33) | 0.83 | 0.77 | 0.86 | 0.84 |
| Catenin beta (Phospho-Ser37) | 0.11 | 0.09 | 0.08 | 0.07 |
| Catenin beta (Phospho-Thr41/Ser45) | 0.81 | 0.80 | 0.98 | 0.93 |
| Catenin beta (Phospho-Tyr489) | 1.49 | 1.81 | 1.74 | 1.35 |
| Catenin beta (Phospho-Tyr654) | 0.96 | 0.78 | 0.90 | 0.95 |
| Catenin delta-1 (Phospho-Tyr228) | 1.09 | 0.98 | 0.92 | 0.89 |
| Caveolin-1 (Phospho-Tyr14) | 0.94 | 0.95 | 0.74 | 1.02 |
| CD19 (Phospho-Tyr531) | 1.01 | 0.84 | 1.42 | 1.50 |
| CD227/mucin 1 (Phospho-Tyr1243) | 1.34 | 1.16 | 1.19 | 1.43 |
| CD3Z (Phospho-Tyr142) | 1.35 | 1.43 | 1.29 | 1.13 |
| CD4 (Phospho-Ser433) | 0.17 | 0.15 | 0.13 | 0.12 |
| CD5 (Phospho-Tyr453) | 0.15 | 0.14 | 0.26 | 0.23 |
| CDC25A (Phospho-Ser124) | 1.70 | 1.82 | 1.20 | 1.12 |
| CDC25A (Phospho-Ser75) | 0.58 | 0.93 | 0.93 | 1.04 |
| CDC25B (Phospho-Ser323) | 0.78 | 0.55 | 0.61 | 0.74 |
| CDC25B (Phospho-Ser353) | 1.49 | 1.38 | 1.08 | 1.09 |
| CDC25C (Phospho-Ser216) | 0.03 | 0.02 | 0.02 | 0.02 |
| CDK1/CDC2 (Phospho-Thr14) | 1.40 | 1.52 | 1.78 | 1.68 |
| CDK1/CDC2 (Phospho-Tyr15) | 1.47 | 2.38 | 1.40 | 1.83 |
| CDK2 (Phospho-Thr160) | 0.75 | 0.79 | 0.61 | 0.63 |
| CDK5 (Phospho-Tyr15) | 0.20 | 0.15 | 0.15 | 0.16 |
| CDK7 (Phospho-Thr170) | 0.83 | 0.70 | 0.70 | 0.59 |
| Chk1 (Phospho-Ser280) | 1.89 | 1.76 | 1.38 | 1.42 |
| Chk1 (Phospho-Ser286) | 0.18 | 0.14 | 0.14 | 0.13 |
| Chk1 (Phospho-Ser296) | 0.31 | 0.28 | 0.26 | 0.24 |
| Chk1 (Phospho-Ser301) | 0.22 | 0.15 | 0.15 | 0.14 |
| Chk1 (Phospho-Ser317) | 0.56 | 0.52 | 0.65 | 0.62 |
| Chk1 (Phospho-Ser345) | 0.59 | 0.57 | 0.52 | 0.62 |
| Chk2 (Phospho-Thr383) | 0.37 | 0.46 | 0.41 | 0.47 |
| Chk2 (Phospho-Thr387) | 0.50 | 0.57 | 0.31 | 0.30 |
| Chk2 (Phospho-Ser516) | 0.60 | 0.61 | 0.56 | 0.41 |
| Chk2 (Phospho-Thr68) | 0.05 | 0.06 | 0.05 | 0.06 |
| c-Jun (Phospho-Tyr170) | 0.97 | 0.92 | 0.95 | 0.78 |
| c-Jun (Phospho-Thr239) | 1.46 | 1.21 | 1.00 | 1.08 |
| c-Jun (Phospho-Ser243) | 0.56 | 0.41 | 0.52 | 0.49 |
| c-Jun (Phospho-Ser63) | 0.95 | 1.26 | 1.22 | 1.13 |
| c-Jun (Phospho-Ser73) | 1.59 | 2.01 | 2.46 | 1.91 |
| c-Jun (Phospho-Thr91) | 1.43 | 1.75 | 1.31 | 1.28 |
| c-Jun (Phospho-Thr93) | 0.49 | 0.64 | 0.54 | 0.32 |
| CK1-A (Phospho-Thr321) | 1.49 | 1.74 | 2.20 | 2.06 |
| CK2-b (Phospho-Ser209) | 0.93 | 0.94 | 0.94 | 0.90 |
| Claudin 3 (Phospho-Tyr219) | 1.63 | 2.07 | 2.55 | 2.62 |
| Claudin 7 (Phospho-Tyr210) | 1.75 | 1.98 | 2.00 | 2.06 |
| Cofilin (Phospho-Ser3) | 0.72 | 0.61 | 0.80 | 0.81 |
| Connexin 43 (Phospho-Ser367) | 0.43 | 0.45 | 0.37 | 0.46 |
| Cortactin (Phospho-Tyr421) | 1.34 | 1.89 | 1.80 | 1.85 |
| Cortactin (Phospho-Tyr466) | 0.63 | 0.87 | 0.70 | 0.66 |
| CPI17 alpha (Phospho-Thr38) | 1.22 | 1.16 | 1.39 | 1.52 |
| c-PLA2 (Phospho-Ser505) | 1.01 | 0.95 | 0.96 | 1.00 |
| CREB (Phospho-Thr100) | 1.31 | 1.42 | 1.47 | 1.36 |
| CREB (Phospho-Ser121) | 1.09 | 1.19 | 0.98 | 0.94 |
| CREB (Phospho-Ser129) | 1.29 | 1.38 | 1.51 | 1.53 |
| CREB (Phospho-Ser133) | 1.15 | 1.11 | 0.99 | 0.89 |
| CREB (Phospho-Ser142) | 0.93 | 0.79 | 0.82 | 0.97 |
| CrkII (Phospho-Tyr221) | 0.72 | 0.27 | 0.39 | 0.29 |
| Cyclin B1 (Phospho-Ser126) | 0.72 | 0.60 | 0.55 | 0.46 |
| Cyclin B1 (Phospho-Ser147) | 1.19 | 1.45 | 1.38 | 1.61 |
| Cyclin D1 (Phospho-Thr286) | 0.31 | 0.64 | 0.28 | 0.13 |
| Cyclin D3 (Phospho-Thr283) | 0.19 | 0.41 | 0.21 | 0.33 |
| Cyclin E1 (Phospho-Thr395) | 0.92 | 0.92 | 0.79 | 0.82 |
| Cyclin E1 (Phospho-Thr77) | 0.12 | 0.15 | 0.09 | 0.14 |
| Cyclin E2 (Phospho-Thr392) | 0.39 | 0.35 | 0.38 | 0.38 |
| DAB1 (Phospho-Tyr220) | 0.98 | 1.22 | 1.43 | 1.40 |
| DAB1 (Phospho-Tyr232) | 1.36 | 1.42 | 1.10 | 1.38 |
| DAPP1 (Phospho-Tyr139) | 0.90 | 0.82 | 1.04 | 0.74 |
| DARPP-32 (Phospho-Thr34) | 0.38 | 0.40 | 0.36 | 0.30 |
| DARPP-32 (Phospho-Thr75) | 0.90 | 0.87 | 0.89 | 0.93 |
| DDX5/DEAD-box protein 5 (Phospho-Tyr593) | 0.83 | 0.78 | 0.83 | 0.67 |
| DNA-PK (Phospho-Thr2638) | 0.47 | 0.45 | 0.40 | 0.37 |
| DNA-PK (Phospho-Thr2647) | 0.63 | 0.50 | 0.69 | 0.68 |
| Dok-1 (Phospho-Tyr362) | 1.09 | 0.82 | 1.01 | 1.33 |
| Dok-1 (Phospho-Tyr398) | 1.18 | 1.00 | 0.99 | 1.25 |
| Dok-2 (Phospho-Tyr299) | 0.90 | 0.68 | 0.82 | 0.71 |
| DYN1 (Phospho-Ser774) | 0.09 | 0.09 | 0.06 | 0.05 |
| E2F1 (Phospho-Thr433) | 0.11 | 0.12 | 0.10 | 0.09 |
| EEF2 (Phospho-Thr56) | 0.42 | 0.39 | 0.34 | 0.31 |
| eEF2K (Phospho-Ser366) | 0.55 | 0.55 | 0.55 | 0.43 |
| EGFR (Phospho-Tyr1016) | 0.89 | 0.94 | 0.84 | 0.82 |
| EGFR (Phospho-Tyr1069) | 1.73 | 1.53 | 1.20 | 1.21 |
| EGFR (Phospho-Ser1070) | 0.05 | 0.04 | 0.04 | 0.05 |
| EGFR (Phospho-Tyr1092) | 0.46 | 0.36 | 0.76 | 0.65 |
| EGFR (Phospho-Tyr1110) | 0.97 | 0.92 | 0.85 | 0.94 |
| EGFR (Phospho-Tyr1172) | 0.54 | 0.53 | 0.41 | 0.40 |
| EGFR (Phospho-Tyr1197) | 0.86 | 0.79 | 0.89 | 0.94 |
| EGFR (Phospho-Thr678) | 1.17 | 1.25 | 1.30 | 1.30 |
| EGFR (Phospho-Thr693) | 0.44 | 0.34 | 0.40 | 0.40 |
| EGFR (Phospho-Tyr869) | 0.06 | 0.07 | 0.06 | 0.05 |
| eIF2A (Phospho-Ser51) | 0.73 | 0.51 | 0.36 | 0.67 |
| eIF4E (Phospho-Ser209) | 1.24 | 1.19 | 1.22 | 1.25 |
| eIF4G (Phospho-Ser1108) | 0.29 | 0.43 | 0.32 | 0.30 |
| Elk1 (Phospho-Ser383) | 1.66 | 1.69 | 2.18 | 2.61 |
| Elk1 (Phospho-Ser389) | 0.80 | 1.02 | 0.80 | 0.79 |
| Elk1 (Phospho-Thr417) | 1.06 | 0.93 | 0.87 | 1.03 |
| eNOS (Phospho-Ser1177) | 1.39 | 1.67 | 1.58 | 2.18 |
| eNOS (Phospho-Thr495) | 1.13 | 1.45 | 1.23 | 1.35 |
| eNOS (Phospho-Ser615) | 0.83 | 0.75 | 0.92 | 0.84 |
| EPB41 (Phospho-Tyr418/660) | 1.15 | 1.32 | 1.31 | 1.15 |
| EPHA2/3/4 (Phospho-Tyr588/596) | 0.97 | 0.75 | 0.87 | 0.91 |
| EPHB1/2 (Phospho-Tyr594/604) | 2.79 | 2.95 | 2.00 | 2.36 |
| Ephrin B1 (Phospho-Tyr317) | 0.70 | 0.63 | 0.72 | 0.76 |
| Ephrin B2 (Phospho-Tyr330) | 0.15 | 0.17 | 0.21 | 0.16 |
| Epo-R (Phospho-Tyr368) | 0.91 | 0.97 | 0.85 | 0.99 |
| ERK3 (Phospho-Ser189) | 1.03 | 0.99 | 0.74 | 0.80 |
| Estrogen Receptor-alpha (Phospho-Ser104) | 0.36 | 0.58 | 0.65 | 0.55 |
| Estrogen Receptor-alpha (Phospho-Ser106) | 0.06 | 0.08 | 0.06 | 0.05 |
| Estrogen Receptor-alpha (Phospho-Ser118) | 2.08 | 2.59 | 2.22 | 2.24 |
| Estrogen Receptor-alpha (Phospho-Ser167) | 1.09 | 1.26 | 1.37 | 0.93 |
| ETK (Phospho-Tyr40) | 1.17 | 1.43 | 1.81 | 1.49 |
| Ezrin (Phospho-Tyr353) | 0.72 | 0.83 | 0.82 | 0.88 |
| Ezrin (Phospho-Tyr478) | 0.64 | 0.67 | 0.51 | 0.64 |
| Ezrin (Phospho-Thr566) | 0.11 | 0.11 | 0.11 | 0.08 |
| FAK (Phospho-Tyr397) | 0.76 | 0.57 | 0.71 | 0.84 |
| FAK (Phospho-Tyr407) | 1.23 | 1.29 | 1.04 | 0.91 |
| FAK (Phospho-Tyr576) | 0.96 | 1.02 | 1.01 | 0.92 |
| FAK (Phospho-Tyr861) | 1.03 | 1.27 | 0.96 | 1.06 |
| FAK (Phospho-Ser910) | 0.08 | 0.05 | 0.08 | 0.09 |
| FAK (Phospho-Tyr925) | 0.73 | 0.63 | 0.73 | 0.84 |
| FAS (Phospho-Tyr291) | 0.68 | 0.88 | 0.73 | 0.73 |
| FER (Phospho-Tyr402) | 2.87 | 2.85 | 2.67 | 3.29 |
| FGFR1 (Phospho-Tyr154) | 1.05 | 0.93 | 0.80 | 0.95 |
| FGFR1 (Phospho-Tyr766) | 0.71 | 0.51 | 0.66 | 0.56 |
| Filamin A (Phospho-Ser2152) | 0.81 | 0.77 | 0.91 | 0.67 |
| FKHR (Phospho-Ser256) | 0.84 | 0.83 | 0.72 | 0.78 |
| FKHR (Phospho-Ser319) | 0.64 | 0.68 | 0.57 | 0.54 |
| FKHR/FOXO1A (Phospho-Ser329) | 1.00 | 0.86 | 0.93 | 0.91 |
| FKHRL1/FOXO3A (Phospho-Ser253) | 0.50 | 0.56 | 0.40 | 0.47 |
| FLT3 (Phospho-Tyr599) | 0.67 | 0.50 | 0.60 | 0.56 |
| Fos (Phospho-Thr232) | 0.69 | 0.77 | 1.02 | 0.93 |
| Fos (Phospho-Ser362) | 0.39 | 0.46 | 0.53 | 0.41 |
| FosB (Phospho-Ser27) | 1.26 | 1.46 | 1.45 | 1.13 |
| FOXO1/3/4-pan (Phospho-Thr24/32) | 0.75 | 0.78 | 0.71 | 0.57 |
| G3BP-1 (Phospho-Ser232) | 0.75 | 0.89 | 0.93 | 0.87 |
| Gab1 (Phospho-Tyr627) | 0.65 | 0.65 | 0.61 | 0.73 |
| Gab1 (Phospho-Tyr659) | 0.74 | 0.86 | 0.80 | 0.93 |
| Gab2 (Phospho-Ser159) | 2.41 | 3.02 | 1.89 | 1.43 |
| Gab2 (Phospho-Tyr643) | 2.45 | 2.26 | 3.95 | 4.65 |
| GABA-RB (Phospho-Ser434) | 1.39 | 1.21 | 1.35 | 1.29 |
| GAP43 (Phospho-Ser41) | 0.43 | 0.30 | 0.30 | 0.33 |
| GATA1 (Phospho-Ser142) | 1.23 | 1.14 | 1.35 | 1.20 |
| GATA1 (Phospho-Ser310) | 0.49 | 0.59 | 0.51 | 0.49 |
| GluR1 (Phospho-Ser849) | 0.02 | 0.02 | 0.01 | 0.01 |
| GluR1 (Phospho-Ser863) | 1.17 | 1.08 | 1.07 | 1.02 |
| GluR2 (Phospho-Ser880) | 0.09 | 0.11 | 0.08 | 0.09 |
| GRB10/Growth factor receptor-bound protein 10 (Phospho-Tyr67) | 1.48 | 1.60 | 1.19 | 1.41 |
| GRK1 (Phospho-Ser21) | 1.07 | 1.26 | 1.36 | 1.05 |
| GRK2 (Phospho-Ser29) | 0.41 | 0.27 | 0.38 | 0.33 |
| GSK3 alpha (Phospho-Ser21) | 1.05 | 1.40 | 1.28 | 1.28 |
| GSK3 alpha/beta (Phospho-Tyr216/279) | 1.76 | 1.64 | 2.01 | 2.06 |
| GSK3 beta (Phospho-Ser9) | 1.63 | 2.55 | 2.05 | 1.76 |
| GTPase activating protein (Phospho-Ser387) | 0.65 | 0.67 | 0.69 | 0.61 |
| HDAC1 (Phospho-Ser421) | 0.09 | 0.03 | 0.08 | 0.03 |
| HDAC2 (Phospho-Ser394) | 1.29 | 1.86 | 1.90 | 0.80 |
| HDAC3 (Phospho-Ser424) | 0.30 | 0.42 | 0.35 | 0.37 |
| HDAC4 (Phospho-Ser632) | 1.43 | 1.33 | 1.38 | 1.30 |
| HDAC5 (Phospho-Ser259) | 1.08 | 1.12 | 0.94 | 0.73 |
| HDAC5 (Phospho-Ser498) | 1.02 | 1.21 | 1.04 | 0.99 |
| HDAC6 (Phospho-Ser22) | 0.17 | 0.18 | 0.14 | 0.13 |
| HDAC8 (Phospho-Ser39) | 0.62 | 0.77 | 0.77 | 0.70 |
| HER2 (Phospho-Tyr1221/Tyr1222) | 0.46 | 0.38 | 0.33 | 0.38 |
| HER2 (Phospho-Tyr1248) | 0.48 | 0.51 | 0.46 | 0.49 |
| HER2 (Phospho-Thr686) | 0.59 | 0.67 | 0.67 | 0.54 |
| HER2 (Phospho-Tyr877) | 1.11 | 1.08 | 0.95 | 1.00 |
| HER3/ErbB3 (Phospho-Tyr1222) | 0.80 | 0.81 | 0.82 | 0.67 |
| HER3/ErbB3 (Phospho-Tyr1289) | 1.01 | 1.28 | 1.18 | 0.97 |
| HER4/ErbB4 (Phospho-Tyr1284) | 0.39 | 0.24 | 0.29 | 0.21 |
| Histone H2A.X (Phospho-Ser139) | 0.29 | 0.37 | 0.25 | 0.34 |
| Histone H3.1 (Phospho-Ser10) | 0.81 | 0.86 | 0.86 | 0.68 |
| HNF4 alpha (Phospho-Ser313) | 0.96 | 1.35 | 0.81 | 1.13 |
| HRS (Phospho-Tyr334) | 1.21 | 1.26 | 1.13 | 1.23 |
| HSF1 (Phospho-Ser303) | 0.93 | 0.91 | 0.94 | 0.98 |
| HSL (Phospho-Ser552/563) | 0.25 | 0.50 | 0.33 | 0.21 |
| HSL (Phospho-Ser554) | 0.62 | 0.60 | 0.66 | 0.55 |
| HSP27 (Phospho-Ser15) | 2.07 | 2.35 | 2.45 | 2.29 |
| HSP27 (Phospho-Ser78) | 1.44 | 1.82 | 1.28 | 1.50 |
| HSP27 (Phospho-Ser82) | 0.88 | 1.20 | 0.79 | 0.97 |
| HSP90 co-chaperone Cdc37 (Phospho-Ser13) | 1.07 | 1.02 | 1.07 | 0.84 |
| HSP90B (Phospho-Ser226) | 2.45 | 1.88 | 3.20 | 2.50 |
| HSP90B (Phospho-Ser254) | 0.88 | 0.91 | 1.39 | 1.06 |
| ICAM-1 (Phospho-Tyr512) | 1.77 | 1.94 | 1.75 | 1.73 |
| IGF1R (Phospho-Tyr1161) | 1.44 | 1.70 | 1.39 | 1.45 |
| IGF1R (Phospho-Tyr1165/1166) | 1.28 | 1.25 | 1.62 | 1.55 |
| IGF2R (Phospho-Ser2409) | 0.98 | 0.87 | 0.83 | 0.84 |
| IkB-alpha (Phospho-Ser32/36) | 13.70 | 11.48 | 13.87 | 12.29 |
| IkB-alpha (Phospho-Tyr42) | 0.84 | 0.85 | 0.84 | 0.81 |
| IkB-beta (Phospho-Ser23) | 0.67 | 0.61 | 0.69 | 0.45 |
| IkB-beta (Phospho-Thr19) | 0.64 | 0.72 | 0.65 | 0.51 |
| IkB-epsilon (Phospho-Ser22) | 0.15 | 0.16 | 0.13 | 0.10 |
| IKK-alpha (Phospho-Thr23) | 0.25 | 0.29 | 0.33 | 0.28 |
| IKK-alpha/beta (Phospho-Ser180/181) | 0.82 | 1.09 | 0.98 | 0.84 |
| IKK-beta (Phospho-Tyr188) | 0.39 | 0.47 | 0.40 | 0.35 |
| IKK-beta (Phospho-Tyr199) | 0.26 | 0.19 | 0.19 | 0.18 |
| IKK-gamma (Phospho-Ser31) | 0.21 | 0.13 | 0.09 | 0.22 |
| IKK-gamma (Phospho-Ser85) | 0.62 | 0.57 | 0.59 | 0.51 |
| IL-10R-alpha (Phospho-Tyr496) | 0.41 | 0.32 | 0.39 | 0.28 |
| IL-13R/CD213a1 (Phospho-Tyr405) | 1.75 | 1.50 | 2.18 | 2.02 |
| IL-2RA/CD25 (Phospho-Ser268) | 1.07 | 0.99 | 0.97 | 0.93 |
| IL3RB (Phospho-Tyr593) | 0.84 | 0.75 | 0.71 | 0.68 |
| IL-4R/CD124 (Phospho-Tyr497) | 0.63 | 0.66 | 0.75 | 1.12 |
| Integrin beta-1 (Phospho-Thr788) | 0.59 | 0.35 | 0.73 | 0.84 |
| Integrin beta-3 (Phospho-Tyr773) | 1.14 | 1.14 | 1.40 | 1.46 |
| Integrin beta-3 (Phospho-Tyr785) | 1.12 | 0.99 | 1.08 | 1.21 |
| Integrin beta-4 (Phospho-Tyr1510) | 1.13 | 1.08 | 1.38 | 1.49 |
| Interferon-alpha/beta receptor alpha chain (Phospho-Tyr466) | 3.44 | 4.65 | 3.62 | 3.48 |
| Interferon-gamma receptor alpha chain precursor (Phospho-Tyr457) | 1.76 | 1.76 | 1.53 | 1.65 |
| IR (Phospho-Tyr1355) | 0.47 | 0.43 | 0.41 | 0.41 |
| IR (Phospho-Tyr1361) | 0.70 | 0.65 | 0.67 | 0.67 |
| IRS-1 (Phospho-Ser307) | 0.92 | 1.07 | 0.91 | 0.93 |
| IRS-1 (Phospho-Ser312) | 0.58 | 0.55 | 0.52 | 0.45 |
| IRS-1 (Phospho-Ser323) | 0.71 | 0.51 | 0.50 | 0.48 |
| IRS-1 (Phospho-Ser636) | 0.88 | 0.85 | 0.81 | 0.80 |
| IRS-1 (Phospho-Ser639) | 0.15 | 0.13 | 0.15 | 0.12 |
| IRS-1 (Phospho-Ser794) | 1.43 | 1.48 | 1.34 | 1.71 |
| JAK1 (Phospho-Tyr1022) | 0.59 | 0.60 | 0.49 | 0.58 |
| JAK2 (Phospho-Tyr1007) | 0.29 | 0.26 | 0.27 | 0.24 |
| JAK2 (Phospho-Tyr221) | 0.78 | 0.85 | 0.84 | 0.70 |
| JNK1/2/3 (Phospho-Thr183/Tyr185) | 1.29 | 1.15 | 1.14 | 1.08 |
| JunB (Phospho-Ser259) | 1.30 | 1.02 | 1.20 | 1.26 |
| JunB (Phospho-Ser79) | 1.09 | 0.80 | 0.89 | 0.97 |
| JunD (Phospho-Ser255) | 1.65 | 1.50 | 1.41 | 1.46 |
| Keratin 18 (Phospho-Ser33) | 0.77 | 0.83 | 0.95 | 1.00 |
| Keratin 18 (Phospho-Ser52) | 2.13 | 1.45 | 1.16 | 1.44 |
| Keratin 8 (Phospho-Ser431) | 0.69 | 0.23 | 0.22 | 0.18 |
| Keratin 8 (Phospho-Ser73) | 0.62 | 0.58 | 0.71 | 0.72 |
| KIT (Phospho-Tyr703) | 0.71 | 0.64 | 0.49 | 0.48 |
| KIT (Phospho-Tyr721) | 0.97 | 0.99 | 1.04 | 1.10 |
| KIT (Phospho-Tyr936) | 1.07 | 0.95 | 1.17 | 0.89 |
| KSR (Phospho-Ser392) | 0.77 | 0.70 | 0.73 | 0.74 |
| Kv1.3/KCNA3 (Phospho-Tyr135) | 0.98 | 1.08 | 1.43 | 1.38 |
| Lamin A (Phospho-Ser22) | 1.12 | 1.04 | 1.25 | 1.36 |
| Lamin A/C (Phospho-Ser392) | 0.03 | 0.02 | 0.02 | 0.02 |
| LAT (Phospho-Tyr171) | 0.50 | 0.48 | 0.38 | 0.45 |
| LAT (Phospho-Tyr191) | 1.21 | 1.09 | 1.32 | 1.01 |
| LCK (Phospho-Tyr192) | 1.25 | 1.14 | 1.15 | 0.99 |
| LCK (Phospho-Tyr393) | 0.12 | 0.09 | 0.20 | 0.17 |
| LCK (Phospho-Tyr504) | 0.88 | 0.94 | 0.79 | 0.76 |
| LCK (Phospho-Ser59) | 0.62 | 0.70 | 0.59 | 0.64 |
| LIMK1 (Phospho-Thr508) | 0.54 | 0.99 | 0.73 | 1.06 |
| LKB1 (Phospho-Thr189) | 1.38 | 1.37 | 1.05 | 1.13 |
| LKB1 (Phospho-Ser428) | 1.89 | 1.17 | 1.48 | 1.78 |
| LYN (Phospho-Tyr507) | 0.09 | 0.08 | 0.06 | 0.11 |
| MAP3K7/TAK1 (Phospho-Thr184) | 0.99 | 0.90 | 0.90 | 0.81 |
| MAP3K8/COT (Phospho-Thr290) | 0.68 | 0.74 | 0.70 | 0.68 |
| MAPKAPK2 (Phospho-Ser272) | 1.11 | 1.19 | 1.62 | 1.30 |
| MARCKS (Phospho-Ser158) | 0.72 | 0.65 | 0.90 | 1.05 |
| MARCKS (Phospho-Ser163) | 1.26 | 1.02 | 1.66 | 1.84 |
| M-CSF Receptor (Phospho-Tyr561) | 1.66 | 1.70 | 2.36 | 1.96 |
| M-CSF Receptor (Phospho-Tyr809) | 1.92 | 2.14 | 2.26 | 2.52 |
| MDM2 (Phospho-Ser166) | 0.03 | 0.03 | 0.02 | 0.02 |
| MEF2A (Phospho-Thr312) | 0.09 | 0.10 | 0.08 | 0.08 |
| MEF2A (Phospho-Thr319) | 0.81 | 0.76 | 0.73 | 0.80 |
| MEF2A (Phospho-Ser408) | 0.36 | 0.39 | 0.43 | 0.47 |
| MEF2C (Phospho-Ser396) | 1.04 | 0.82 | 0.88 | 1.19 |
| MEK1 (Phospho-Ser217) | 1.66 | 1.65 | 2.01 | 1.33 |
| MEK1 (Phospho-Ser221) | 3.43 | 3.24 | 2.81 | 2.93 |
| MEK1 (Phospho-Thr286) | 0.77 | 0.39 | 1.28 | 1.61 |
| MEK1 (Phospho-Thr291) | 0.23 | 0.24 | 0.22 | 0.23 |
| MEK1 (Phospho-Ser298) | 0.43 | 0.39 | 0.32 | 0.16 |
| MEK2 (Phospho-Thr394) | 2.02 | 1.85 | 2.34 | 2.35 |
| Merlin (Phospho-Ser10) | 0.07 | 0.06 | 0.06 | 0.08 |
| Merlin (Phospho-Ser518) | 1.32 | 1.37 | 1.15 | 1.36 |
| Met (Phospho-Tyr1003) | 0.98 | 0.74 | 0.73 | 0.69 |
| Met (Phospho-Tyr1234) | 1.31 | 0.78 | 1.46 | 1.40 |
| Met (Phospho-Tyr1349) | 1.01 | 0.91 | 0.79 | 1.02 |
| Met (Phospho-Tyr1356) | 11.24 | 8.01 | 9.96 | 9.94 |
| MITF (Phospho-Ser73) | 0.31 | 0.28 | 0.39 | 0.30 |
| MKK3/MAP2K3 (Phospho-Ser189) | 0.93 | 0.54 | 0.65 | 0.74 |
| MKK3/MAP2K3 (Phospho-Thr222) | 0.62 | 0.80 | 0.78 | 0.69 |
| MKK4/SEK1 (Phospho-Ser257) | 1.01 | 0.94 | 0.86 | 0.79 |
| MKK4/SEK1 (Phospho-Thr261) | 0.11 | 0.12 | 0.08 | 0.07 |
| MKK4/SEK1 (Phospho-Ser80) | 0.11 | 0.12 | 0.10 | 0.11 |
| MKK6/MAP2K6 (Phospho-Ser207) | 0.71 | 0.89 | 0.67 | 0.73 |
| MKK7/MAP2K7 (Phospho-Ser271) | 1.07 | 1.00 | 0.98 | 1.08 |
| MKK7/MAP2K7 (Phospho-Thr275) | 1.27 | 1.41 | 1.45 | 1.48 |
| MKP-1 (Phospho-Ser359) | 0.75 | 0.79 | 0.74 | 0.57 |
| MKP-1/2 (Phospho-Ser296/318) | 0.60 | 0.52 | 0.43 | 0.53 |
| Mnk1 (Phospho-Thr385) | 1.41 | 1.61 | 1.97 | 1.57 |
| MSK1 (Phospho-Ser360) | 0.24 | 0.26 | 0.14 | 0.19 |
| MSK1 (Phospho-Ser376) | 1.62 | 1.93 | 1.89 | 1.71 |
| MSK1 (Phospho-Thr581) | 0.65 | 0.65 | 0.52 | 0.61 |
| Mst1/Mst2 (Phospho-Thr183) | 0.63 | 0.79 | 0.66 | 0.69 |
| mTOR (Phospho-Thr2446) | 2.42 | 2.59 | 2.04 | 2.20 |
| mTOR (Phospho-Ser2448) | 1.34 | 1.26 | 1.27 | 1.33 |
| mTOR (Phospho-Ser2481) | 0.83 | 0.72 | 0.70 | 0.85 |
| Myc (Phospho-Thr358) | 0.07 | 0.06 | 0.05 | 0.05 |
| Myc (Phospho-Ser373) | 0.76 | 0.78 | 0.70 | 0.60 |
| Myc (Phospho-Thr58) | 0.69 | 0.70 | 0.64 | 0.68 |
| Myc (Phospho-Ser62) | 0.74 | 0.57 | 0.83 | 0.78 |
| Myosin regulatory light chain 2 (Phospho-Ser18) | 0.64 | 0.48 | 0.74 | 0.74 |
| NFAT4 (Phospho-Ser165) | 1.01 | 0.75 | 1.10 | 1.11 |
| NFkB-p100/p52 (Phospho-Ser865) | 0.23 | 0.22 | 0.23 | 0.24 |
| NFkB-p100/p52 (Phospho-Ser869) | 0.62 | 0.73 | 0.96 | 0.88 |
| NFkB-p100/p52 (Phospho-Ser872) | 1.82 | 2.53 | 2.38 | 2.60 |
| NFkB-p105/p50 (Phospho-Ser337) | 1.36 | 2.00 | 1.52 | 1.60 |
| NFkB-p105/p50 (Phospho-Ser893) | 0.69 | 0.87 | 0.69 | 0.67 |
| NFkB-p105/p50 (Phospho-Ser907) | 1.04 | 1.15 | 0.90 | 1.08 |
| NFkB-p105/p50 (Phospho-Ser927) | 0.31 | 0.29 | 0.17 | 0.15 |
| NFkB-p105/p50 (Phospho-Ser932) | 1.00 | 1.00 | 0.93 | 1.04 |
| NFkB-p65 (Phospho-Thr254) | 1.39 | 1.93 | 1.47 | 1.28 |
| NFkB-p65 (Phospho-Ser276) | 1.08 | 1.65 | 1.24 | 1.10 |
| NFkB-p65 (Phospho-Ser311) | 2.14 | 2.20 | 2.85 | 2.86 |
| NFkB-p65 (Phospho-Thr435) | 1.38 | 1.26 | 1.52 | 1.56 |
| NFkB-p65 (Phospho-Ser468) | 2.28 | 2.06 | 1.57 | 3.16 |
| NFkB-p65 (Phospho-Thr505) | 0.36 | 0.29 | 0.42 | 0.37 |
| NFkB-p65 (Phospho-Ser529) | 0.84 | 0.75 | 0.70 | 0.71 |
| NFkB-p65 (Phospho-Ser536) | 1.08 | 1.09 | 1.53 | 1.37 |
| NMDAR1 (Phospho-Ser897) | 1.21 | 1.19 | 1.06 | 1.15 |
| NMDAR2B (Phospho-Tyr1472) | 0.15 | 0.14 | 0.18 | 0.15 |
| Opioid Receptor (Phospho-Ser375) | 2.54 | 2.90 | 2.34 | 2.67 |
| p130Cas (Phospho-Tyr165) | 1.23 | 1.20 | 1.03 | 1.06 |
| p130Cas (Phospho-Tyr410) | 1.02 | 0.68 | 1.11 | 0.90 |
| p21Cip1 (Phospho-Thr145) | 0.72 | 0.79 | 1.03 | 1.04 |
| p27Kip1 (Phospho-Ser10) | 1.66 | 1.83 | 1.74 | 1.66 |
| p27Kip1 (Phospho-Thr187) | 1.64 | 1.66 | 2.14 | 1.72 |
| p38 MAPK (Phospho-Thr180) | 1.34 | 1.45 | 1.16 | 1.20 |
| p38 MAPK (Phospho-Tyr182) | 0.05 | 0.09 | 0.06 | 0.08 |
| p38 MAPK (Phospho-Tyr322) | 0.68 | 0.64 | 0.68 | 0.85 |
| p44/42 MAPK (Phospho-Thr202) | 1.55 | 1.68 | 1.70 | 1.60 |
| p44/42 MAPK (Phospho-Tyr204) | 1.77 | 1.86 | 1.54 | 2.01 |
| p53 (Phospho-Ser15) | 1.66 | 1.54 | 1.77 | 1.60 |
| p53 (Phospho-Thr18) | 0.20 | 0.19 | 0.22 | 0.26 |
| p53 (Phospho-Ser20) | 0.37 | 0.33 | 0.33 | 0.32 |
| p53 (Phospho-Ser315) | 0.15 | 0.16 | 0.06 | 0.16 |
| p53 (Phospho-Ser33) | 0.49 | 0.39 | 0.48 | 0.52 |
| p53 (Phospho-Ser37) | 0.69 | 0.58 | 0.71 | 0.86 |
| p53 (Phospho-Ser366) | 0.93 | 0.98 | 1.03 | 1.02 |
| p53 (Phospho-Ser378) | 1.54 | 1.63 | 1.28 | 1.32 |
| p53 (Phospho-Ser392) | 0.80 | 0.74 | 0.95 | 0.74 |
| p53 (Phospho-Ser46) | 1.15 | 1.06 | 1.25 | 1.47 |
| p53 (Phospho-Ser6) | 1.28 | 1.03 | 1.64 | 1.48 |
| p53 (Phospho-Ser9) | 0.70 | 0.72 | 0.69 | 0.61 |
| P70S6K (Phospho-Thr229) | 0.57 | 0.56 | 0.58 | 0.57 |
| P70S6K (Phospho-Ser371) | 0.58 | 0.79 | 0.85 | 0.81 |
| P70S6K (Phospho-Ser411) | 0.96 | 1.43 | 1.13 | 1.06 |
| P70S6K (Phospho-Thr389) | 1.24 | 1.43 | 1.16 | 1.13 |
| P70S6K (Phospho-Ser418) | 0.92 | 1.07 | 1.07 | 0.99 |
| P70S6K (Phospho-Thr421) | 0.85 | 0.86 | 0.70 | 0.64 |
| P70S6K (Phospho-Ser424) | 1.02 | 1.14 | 1.04 | 1.15 |
| P70S6K-beta (Phospho-Ser423) | 0.55 | 0.65 | 0.48 | 0.49 |
| P73 (Phospho-Tyr99) | 0.91 | 1.25 | 1.04 | 1.02 |
| P90RSK (Phospho-Thr359/Ser363) | 0.14 | 0.06 | 0.05 | 0.12 |
| P90RSK (Phospho-Ser380) | 0.87 | 1.12 | 0.98 | 0.93 |
| P90RSK (Phospho-Thr573) | 0.68 | 0.79 | 0.75 | 0.80 |
| P95/NBS1 (Phospho-Ser343) | 0.27 | 0.32 | 0.23 | 0.24 |
| PAK1 (Phospho-Ser204) | 0.91 | 1.20 | 1.42 | 1.34 |
| PAK1 (Phospho-Thr212) | 0.78 | 0.74 | 0.77 | 1.00 |
| PAK1/2 (Phospho-Ser199) | 0.92 | 0.90 | 0.91 | 0.87 |
| PAK1/2/3 (Phospho-Ser141) | 0.80 | 0.86 | 0.81 | 0.83 |
| PAK1/2/3 (Phospho-Thr423/402/421) | 0.02 | 0.02 | 0.02 | 0.01 |
| PAK2 (Phospho-Ser192) | 0.81 | 0.87 | 1.03 | 0.99 |
| PAK3 (Phospho-Ser154) | 0.80 | 0.90 | 0.98 | 1.29 |
| Paxillin (Phospho-Tyr118) | 1.34 | 1.60 | 1.24 | 1.03 |
| Paxillin (Phospho-Tyr31) | 0.64 | 1.14 | 0.67 | 0.49 |
| PDGFR alpha (Phospho-Tyr849) | 0.90 | 0.62 | 0.74 | 0.68 |
| PDGFR beta (Phospho-Tyr1021) | 1.98 | 1.74 | 2.59 | 2.30 |
| PDGFR beta (Phospho-Tyr740) | 1.61 | 1.28 | 1.26 | 1.36 |
| PDGFR beta (Phospho-Tyr751) | 0.56 | 0.38 | 0.49 | 0.53 |
| PDK1 (Phospho-Ser241) | 0.20 | 0.22 | 0.24 | 0.20 |
| PEA-15 (Phospho-Ser104) | 0.96 | 1.02 | 1.04 | 1.22 |
| PEA-15 (Phospho-Ser116) | 0.85 | 1.00 | 0.91 | 0.90 |
| PECAM-1 (Phospho-Tyr713) | 0.04 | 0.03 | 0.03 | 0.03 |
| PI3-kinase p85-subunit alpha/gamma (Phospho-Tyr467/Tyr199) | 1.16 | 1.17 | 1.11 | 0.91 |
| Pim-1 (Phospho-Tyr309) | 0.70 | 0.82 | 1.10 | 1.02 |
| PKA CAT (Phospho-Thr197) | 0.63 | 0.68 | 0.68 | 0.61 |
| PKC alpha (Phospho-Tyr657) | 1.28 | 1.28 | 1.13 | 1.25 |
| PKC alpha/beta II (Phospho-Thr638) | 0.94 | 0.87 | 0.78 | 0.95 |
| PKC beta/PKCB (Phospho-Ser661) | 0.71 | 0.96 | 0.95 | 1.06 |
| PKC delta (Phospho-Thr505) | 1.44 | 1.60 | 1.27 | 1.33 |
| PKC delta (Phospho-Ser645) | 0.65 | 0.58 | 0.47 | 0.68 |
| PKC epsilon (Phospho-Ser729) | 0.99 | 0.82 | 0.79 | 1.01 |
| PKC pan activation site (Phospho) | 0.69 | 0.58 | 0.85 | 0.76 |
| PKC theta (Phospho-Ser676) | 1.30 | 0.99 | 1.25 | 1.25 |
| PKC zeta (Phospho-Thr410) | 0.67 | 0.84 | 0.65 | 0.62 |
| PKC zeta (Phospho-Thr560) | 0.97 | 0.90 | 1.07 | 1.13 |
| PKD1/PKC mu (Phospho-Ser205) | 0.96 | 0.79 | 0.82 | 0.85 |
| PKD1/PKC mu (Phospho-Tyr463) | 0.62 | 0.51 | 0.53 | 0.55 |
| PKD1/PKC mu (Phospho-Ser910) | 1.20 | 1.09 | 1.01 | 1.05 |
| PKD2 (Phospho-Ser876) | 2.32 | 2.06 | 1.99 | 2.13 |
| PKR (Phospho-Thr446) | 1.29 | 1.23 | 1.23 | 1.32 |
| PKR (Phospho-Thr451) | 0.75 | 0.79 | 0.79 | 0.97 |
| PLC beta3 (Phospho-Ser1105) | 0.90 | 0.79 | 0.73 | 0.67 |
| PLC beta3 (Phospho-Ser537) | 1.22 | 1.15 | 1.34 | 1.23 |
| PLCG1 (Phospho-Tyr771) | 0.82 | 0.69 | 1.03 | 1.24 |
| PLCG1 (Phospho-Tyr783) | 0.73 | 0.54 | 0.60 | 0.75 |
| PLCG2 (Phospho-Tyr1217) | 0.79 | 0.72 | 0.84 | 0.90 |
| PLCG2 (Phospho-Tyr753) | 0.19 | 0.49 | 0.27 | 0.18 |
| PLD1 (Phospho-Ser561) | 0.65 | 0.99 | 0.82 | 0.72 |
| PLK1 (Phospho-Thr210) | 0.08 | 0.05 | 0.08 | 0.07 |
| PP1 alpha (Phospho-Thr320) | 0.70 | 0.66 | 0.75 | 0.71 |
| PP2A-alpha (Phospho-Tyr307) | 0.09 | 0.09 | 0.07 | 0.08 |
| PPAR-BP (Phospho-Thr1457) | 1.11 | 0.96 | 0.96 | 1.04 |
| PPAR-gamma (Phospho-Ser112) | 0.01 | 0.01 | 0.01 | 0.01 |
| Progesterone Receptor (Phospho-Ser190) | 0.88 | 1.14 | 0.66 | 1.10 |
| PTEN (Phospho-Ser370) | 0.20 | 0.22 | 0.23 | 0.25 |
| PTEN (Phospho-Ser380) | 0.27 | 0.31 | 0.30 | 0.24 |
| PTEN (Phospho-Ser380/Thr382/Thr383) | 0.38 | 0.42 | 0.35 | 0.38 |
| Pyk2 (Phospho-Tyr402) | 1.18 | 0.88 | 0.96 | 0.93 |
| Pyk2 (Phospho-Tyr579) | 0.52 | 0.54 | 0.44 | 0.51 |
| Pyk2 (Phospho-Tyr580) | 0.49 | 0.44 | 0.36 | 0.43 |
| Pyk2 (Phospho-Tyr881) | 0.34 | 0.17 | 0.18 | 0.15 |
| Rac1/cdc42 (Phospho-Ser71) | 1.12 | 1.40 | 1.40 | 1.36 |
| RAD51 (Phospho-Tyr315) | 0.79 | 0.82 | 0.62 | 0.64 |
| Raf1 (Phospho-Ser259) | 1.30 | 1.38 | 1.05 | 2.08 |
| Raf1 (Phospho-Ser289) | 0.50 | 0.57 | 0.65 | 0.60 |
| Raf1 (Phospho-Ser296) | 0.86 | 1.08 | 1.01 | 1.12 |
| Raf1 (Phospho-Ser338) | 0.92 | 1.22 | 1.48 | 1.06 |
| Raf1 (Phospho-Tyr341) | 1.22 | 1.40 | 1.24 | 1.25 |
| Raf1 (Phospho-Ser43) | 0.21 | 0.25 | 0.25 | 0.18 |
| Raf1 (Phospho-Ser621) | 1.53 | 1.71 | 1.37 | 1.54 |
| Ras-GRF1 (Phospho-Ser916) | 0.24 | 0.53 | 0.35 | 0.34 |
| Rb (Phospho-Ser608) | 1.26 | 1.37 | 1.51 | 1.62 |
| Rb (Phospho-Ser780) | 0.20 | 0.22 | 0.20 | 0.20 |
| Rb (Phospho-Ser795) | 0.13 | 0.14 | 0.12 | 0.13 |
| Rb (Phospho-Ser807) | 0.36 | 0.34 | 0.36 | 0.37 |
| Rb (Phospho-Ser811) | 0.56 | 0.43 | 0.55 | 0.58 |
| Rb (Phospho-Thr821) | 0.54 | 0.45 | 0.47 | 0.46 |
| Rel (Phospho-Ser503) | 1.08 | 0.88 | 0.92 | 0.83 |
| RelB (Phospho-Ser552) | 0.84 | 0.98 | 0.78 | 0.96 |
| Ret (Phospho-Tyr905) | 1.47 | 1.65 | 1.65 | 1.68 |
| Rho/Rac guanine nucleotide exchange factor 2 (Phospho-Ser885) | 0.63 | 0.64 | 0.68 | 0.80 |
| RSK1/2/3/4 (Phospho-Ser221/227/218/232) | 0.45 | 0.69 | 0.71 | 0.71 |
| RyR2 (Phospho-Ser2808) | 0.91 | 1.31 | 1.25 | 1.17 |
| S6 Ribosomal Protein (Phospho-Ser235) | 1.15 | 1.22 | 1.11 | 1.05 |
| SAPK/JNK (Phospho-Thr183) | 0.99 | 1.07 | 0.92 | 0.91 |
| SAPK/JNK (Phospho-Tyr185) | 1.26 | 1.47 | 1.10 | 1.48 |
| Shc (Phospho-Tyr349) | 0.09 | 0.13 | 0.13 | 0.13 |
| Shc (Phospho-Tyr427) | 0.35 | 0.26 | 0.19 | 0.27 |
| SHP-2 (Phospho-Tyr542) | 1.12 | 1.15 | 1.03 | 1.34 |
| SHP-2 (Phospho-Tyr580) | 0.99 | 0.97 | 0.96 | 0.97 |
| SLP-76 (Phospho-Tyr128) | 1.28 | 1.43 | 1.81 | 1.85 |
| Smad1 (Phospho-Ser187) | 1.30 | 1.05 | 1.35 | 1.17 |
| Smad1 (Phospho-Ser465) | 0.99 | 0.71 | 1.00 | 0.75 |
| Smad2 (Phospho-Thr220) | 0.58 | 0.64 | 0.63 | 0.55 |
| Smad2 (Phospho-Ser250) | 0.94 | 0.88 | 1.23 | 0.98 |
| Smad2 (Phospho-Ser467) | 0.75 | 0.78 | 0.76 | 0.92 |
| Smad2/3 (Phospho-Thr8) | 0.83 | 0.85 | 0.81 | 0.75 |
| Smad3 (Phospho-Thr179) | 0.59 | 0.63 | 0.56 | 0.49 |
| Smad3 (Phospho-Ser204) | 0.91 | 1.02 | 1.08 | 0.81 |
| Smad3 (Phospho-Ser208) | 1.12 | 1.31 | 1.35 | 1.13 |
| Smad3 (Phospho-Ser213) | 1.95 | 2.50 | 2.15 | 1.72 |
| Smad3 (Phospho-Ser425) | 0.77 | 0.78 | 0.83 | 0.69 |
| SMC1 (Phospho-Ser957) | 1.36 | 1.68 | 1.79 | 1.64 |
| SP1 (Phospho-Thr739) | 1.33 | 1.77 | 1.36 | 1.61 |
| Src (Phospho-Tyr418) | 0.05 | 0.04 | 0.03 | 0.03 |
| Src (Phospho-Tyr529) | 0.83 | 0.74 | 0.81 | 0.78 |
| Src (Phospho-Ser75) | 1.37 | 1.80 | 1.35 | 1.56 |
| SREBP-1 (Phospho-Ser439) | 0.76 | 0.66 | 0.76 | 0.76 |
| SRF (Phospho-Ser77) | 0.94 | 1.25 | 1.09 | 0.88 |
| SRF (Phospho-Ser99) | 0.17 | 0.14 | 0.15 | 0.17 |
| STAM2 (Phospho-Tyr192) | 1.77 | 1.77 | 2.08 | 2.72 |
| STAT1 (Phospho-Tyr701) | 1.82 | 1.77 | 1.69 | 1.60 |
| STAT1 (Phospho-Ser727) | 1.14 | 1.29 | 1.26 | 1.33 |
| STAT2 (Phospho-Tyr690) | 0.85 | 0.70 | 0.72 | 0.69 |
| STAT3 (Phospho-Tyr705) | 0.69 | 0.78 | 0.57 | 0.54 |
| STAT3 (Phospho-Ser727) | 0.84 | 0.89 | 0.89 | 0.87 |
| STAT4 (Phospho-Tyr693) | 1.12 | 1.37 | 1.05 | 1.04 |
| STAT5A (Phospho-Tyr694) | 1.26 | 1.31 | 1.01 | 1.04 |
| STAT5A (Phospho-Ser780) | 0.07 | 0.08 | 0.05 | 0.06 |
| STAT5B (Phospho-Ser731) | 0.79 | 0.69 | 0.75 | 0.77 |
| STAT6 (Phospho-Tyr641) | 1.32 | 1.58 | 1.17 | 1.19 |
| STAT6 (Phospho-Thr645) | 0.19 | 0.17 | 0.13 | 0.13 |
| Stathmin 1 (Phospho-Ser15) | 1.01 | 1.01 | 0.72 | 0.76 |
| Stathmin 1 (Phospho-Ser24) | 1.39 | 1.37 | 1.16 | 1.19 |
| Stathmin 1 (Phospho-Ser37) | 0.84 | 0.71 | 0.85 | 0.85 |
| Survivin (Phospho-Thr117) | 0.85 | 0.77 | 0.78 | 0.70 |
| SYK (Phospho-Tyr348) | 0.62 | 0.64 | 0.51 | 0.53 |
| SYK (Phospho-Tyr525) | 0.22 | 0.16 | 0.12 | 0.11 |
| Synapsin (Phospho-Ser62) | 0.72 | 0.98 | 0.86 | 0.95 |
| Synapsin (Phospho-Ser9) | 0.80 | 0.81 | 0.78 | 0.90 |
| Synaptotagmin (Phospho-Thr202) | 0.94 | 1.00 | 0.88 | 0.94 |
| Synaptotagmin (Phospho-Ser309) | 0.90 | 1.28 | 1.13 | 1.39 |
| Synuclein alpha (Phospho-Tyr125) | 0.63 | 0.71 | 0.65 | 0.86 |
| Synuclein alpha (Phospho-Tyr133) | 0.90 | 0.76 | 0.82 | 0.89 |
| Synuclein alpha (Phospho-Tyr136) | 0.69 | 0.74 | 0.69 | 0.89 |
| Tau (Phospho-Thr181) | 1.00 | 1.17 | 1.13 | 1.11 |
| Tau (Phospho-Thr205) | 0.66 | 0.61 | 0.64 | 0.67 |
| Tau (Phospho-Thr212) | 0.11 | 0.09 | 0.06 | 0.06 |
| Tau (Phospho-Ser214) | 0.41 | 0.47 | 0.50 | 0.55 |
| Tau (Phospho-Thr231) | 0.98 | 1.22 | 1.33 | 1.31 |
| Tau (Phospho-Ser235) | 0.10 | 0.11 | 0.08 | 0.09 |
| Tau (Phospho-Ser262) | 0.73 | 0.75 | 0.88 | 0.87 |
| Tau (Phospho-Ser356) | 0.05 | 0.05 | 0.03 | 0.03 |
| Tau (Phospho-Ser396) | 0.27 | 0.28 | 0.34 | 0.37 |
| Tau (Phospho-Ser404) | 0.82 | 0.81 | 0.95 | 0.95 |
| Tau (Phospho-Ser422) | 1.03 | 1.12 | 1.15 | 1.06 |
| TIF-IA (Phospho-Ser649) | 1.27 | 1.31 | 1.33 | 1.30 |
| TOP2A/DNA topoisomerase II (Phospho-Ser1106) | 0.30 | 0.17 | 0.22 | 0.19 |
| Trk B (Phospho-Tyr515) | 0.51 | 0.61 | 0.52 | 0.47 |
| Tuberin/TSC2 (Phospho-Thr1462) | 1.73 | 2.19 | 1.57 | 1.34 |
| Tuberin/TSC2 (Phospho-Ser939) | 0.40 | 0.55 | 0.64 | 0.62 |
| TYK2 (Phospho-Tyr1054) | 0.82 | 0.71 | 0.60 | 0.57 |
| Tyrosine Hydroxylase (Phospho-Ser19) | 0.71 | 0.73 | 0.57 | 0.56 |
| Tyrosine Hydroxylase (Phospho-Ser31) | 0.73 | 0.64 | 0.71 | 0.66 |
| Tyrosine Hydroxylase (Phospho-Ser40) | 0.48 | 0.44 | 0.50 | 0.36 |
| Tyrosine Hydroxylase (Phospho-Ser8) | 0.95 | 1.07 | 1.07 | 1.05 |
| VASP (Phospho-Ser157) | 0.11 | 0.10 | 0.12 | 0.13 |
| VASP (Phospho-Ser238) | 2.30 | 2.55 | 1.15 | 1.92 |
| VAV1 (Phospho-Tyr174) | 0.93 | 0.96 | 0.84 | 0.86 |
| VAV2 (Phospho-Tyr142) | 1.85 | 2.17 | 2.43 | 2.19 |
| VEGFR1 (Phospho-Tyr1333) | 1.33 | 1.86 | 1.44 | 1.44 |
| VEGFR2 (Phospho-Tyr1054) | 0.80 | 0.75 | 1.01 | 0.95 |
| VEGFR2 (Phospho-Tyr1059) | 1.24 | 1.57 | 1.12 | 1.26 |
| VEGFR2 (Phospho-Tyr1175) | 1.00 | 0.89 | 0.81 | 1.15 |
| VEGFR2 (Phospho-Tyr1214) | 0.09 | 0.09 | 0.07 | 0.07 |
| VEGFR2 (Phospho-Tyr951) | 0.10 | 0.09 | 0.10 | 0.10 |
| Vinculin (Phospho-Tyr821) | 1.57 | 1.46 | 1.75 | 1.46 |
| WASP (Phospho-Tyr290) | 0.45 | 0.53 | 1.22 | 1.38 |
| WAVE1 (Phospho-Tyr125) | 0.99 | 0.88 | 0.98 | 1.00 |
| WEE1 (Phospho-Ser53) | 0.35 | 0.32 | 0.38 | 0.36 |
| XIAP (Phospho-Ser87) | 0.45 | 0.47 | 0.43 | 0.40 |
| Zap-70 (Phospho-Tyr292) | 1.02 | 1.20 | 1.20 | 1.22 |
| Zap-70 (Phospho-Tyr315) | 0.55 | 0.51 | 0.55 | 0.60 |
| Zap-70 (Phospho-Tyr319) | 1.06 | 0.92 | 0.95 | 0.92 |
| Zap-70 (Phospho-Tyr493) | 1.20 | 1.34 | 1.21 | 1.75 |

**Supplementary table 4**

List of altered phospho-sites (30% decreased or 50% increased phosphorylation) from phosphoproteome analysis of U343-MG cells 1 h after indicated treatment in comparison to the control IgG/DMSO treatment as shown in Fig. 4A.

| **phosphorylation sites** |
| --- |
| 1:WASP (Phospho-Tyr290) |
| 2:HSL (Phospho-Ser552/563) |
| 3:Paxillin (Phospho-Tyr31) |
| 4:BLNK (Phospho-Tyr96) |
| 5:BLNK (Phospho-Tyr96) |
| 6:Cyclin D1 (Phospho-Thr286) |
| 7:Cyclin D1 (Phospho-Thr286) |
| 8:NFkB-p65 (Phospho-Ser276) |
| 9:P70S6K (Phospho-Ser411) |
| 10:GSK3 beta (Phospho-Ser9) |
| 11:Abl1 (Phospho-Tyr204) |
| 12:PLD1 (Phospho-Ser561) |
| 13:14-3-3 zeta (Phospho-Ser58) |
| 14:CDK1/CDC2 (Phospho-Tyr15) |
| 15:c-Jun (Phospho-Thr93) |
| 16:HDAC2 (Phospho-Ser394) |
| 17:p53 (Phospho-Ser315) |
| 18:Raf1 (Phospho-Ser259) |
| 19:LYN (Phospho-Tyr507) |
| 20:NFkB-p65 (Phospho-Ser468) |
| 21:HDAC1 (Phospho-Ser421) |
| 22:Keratin 8 (Phospho-Ser431) |
| 23:CrkII (Phospho-Tyr221) |
| 24:Pyk2 (Phospho-Tyr881) |
| 25:Caspase 9 (Phospho-Tyr153) |
| 26:P90RSK (Phospho-Thr359/Ser363) |
| 27:eIF2A (Phospho-Ser51) |
| 28:IKK-gamma (Phospho-Ser31) |
| 29:FAK (Phospho-Ser910) |
| 30:Met (Phospho-Tyr1234) |
| 31:MKK3/MAP2K3 (Phospho-Ser189) |
| 32:BTK (Phospho-Tyr223) |
| 33:LKB1 (Phospho-Ser428) |
| 34:PDGFR beta (Phospho-Tyr751) |
| 35:IL-10R-alpha (Phospho-Tyr496) |
| 36:p130Cas (Phospho-Tyr410) |
| 37:GRK2 (Phospho-Ser29) |
| 38:PLK1 (Phospho-Thr210) |
| 39:Tau (Phospho-Ser356) |
| 40:MSK1 (Phospho-Ser360) |
| 41:VASP (Phospho-Ser238) |
| 42:BLNK (Phospho-Tyr84) |
| 43:Gab2 (Phospho-Ser159) |
| 44:ALK (Phospho-Tyr1604) |
| 45:AMPK beta1 (Phospho-Ser182) |
| 46:Chk2 (Phospho-Ser516) |
| 47:HDAC5 (Phospho-Ser259) |
| 48:IkB-epsilon (Phospho-Ser22) |
| 49:Chk2 (Phospho-Thr387) |
| 50:DYN1 (Phospho-Ser774) |
| 51:MDM2 (Phospho-Ser166) |
| 52:ACC1 (Phospho-Ser80) |
| 53:PAK1/2/3 (Phospho-Thr423/402/421) |
| 54:CDC25A (Phospho-Ser124) |
| 55:MKK4/SEK1 (Phospho-Thr261) |
| 56:MEK1 (Phospho-Ser298) |
| 57:Caspase 9 (Phospho-Ser196) |
| 58:SYK (Phospho-Tyr525) |
| 59:TOP2A/DNA topoisomerase II (Phospho-Ser1106) |
| 60:ASK1 (Phospho-Ser966) |
| 61:HER4/ErbB4 (Phospho-Tyr1284) |
| 62:NFkB-p105/p50 (Phospho-Ser927) |
| 63:GluR1 (Phospho-Ser849) |
| 64:Src (Phospho-Tyr418) |
| 65:Tau (Phospho-Thr212) |
| 66:Keratin 18 (Phospho-Ser52) |
| 67:Shc (Phospho-Tyr427) |
| 68:Cyclin B1 (Phospho-Ser126) |
| 69:c-Jun (Phospho-Thr239) |
| 70:PPAR-gamma (Phospho-Ser112) |
| 71:EGFR (Phospho-Tyr1069) |
| 72:KIT (Phospho-Tyr703) |
| 73:STAT6 (Phospho-Thr645) |
| 74:Catenin beta (Phospho-Ser37) |
| 75:TYK2 (Phospho-Tyr1054) |
| 76:PDGFR alpha (Phospho-Tyr849) |
| 77:IKK-beta (Phospho-Tyr199) |
| 78:Chk1 (Phospho-Ser301) |
| 79:IRS-1 (Phospho-Ser323) |
| 80:AKT1 (Phospho-Ser124) |
| 81:ATF2 (Phospho-Thr71/53) |
| 82:CDC25C (Phospho-Ser216) |
| 83:GAP43 (Phospho-Ser41) |
| 84:Integrin beta-1 (Phospho-Thr788) |
| 85:Estrogen Receptor-alpha (Phospho-Ser104) |
| 86:CDC25A (Phospho-Ser75) |
| 87:RSK1/2/3/4 (Phospho-Ser221/227/218/232) |
| 88:Shc (Phospho-Tyr349) |
| 89:Synaptotagmin (Phospho-Ser309) |
| 90:PLCG1 (Phospho-Tyr771) |
| 91:Elk1 (Phospho-Ser383) |
| 92:STAM2 (Phospho-Tyr192) |
| 93:IL-4R/CD124 (Phospho-Tyr497) |
| 94:eNOS (Phospho-Ser1177) |
| 95:PAK3 (Phospho-Ser154) |
| 96:CD5 (Phospho-Tyr453) |
| 97:EGFR (Phospho-Tyr1092) |
| 98:LCK (Phospho-Tyr393) |
| 99:c-Jun (Phospho-Ser73) |
| 100:ETK (Phospho-Tyr40) |
| 101:BCR (Phospho-Tyr360) |
| 102:ALK (Phospho-Tyr1507) |
| 103:HSP90B (Phospho-Ser254) |
| 104:Amyloid beta A4 (Phospho-Thr743/668) |
| 105:NFkB-p100/p52 (Phospho-Ser869) |
| 106:Pim-1 (Phospho-Tyr309) |
| 107:Claudin 3 (Phospho-Tyr219) |
| 108:PAK1 (Phospho-Ser204) |
| 109:Tuberin/TSC2 (Phospho-Ser939) |
| 110:4E-BP1 (Phospho-Ser65) |
| 111:MEK1 (Phospho-Thr286) |
| 112:MEK1 (Phospho-Thr286) |
| 113:Catalase (Phospho-Tyr385) |
| 114:Gab2 (Phospho-Tyr643) |
| 115:Cyclin D3 (Phospho-Thr283) |
| 116:p38 MAPK (Phospho-Tyr182) |
| 117:Actin Pan (a/b/g) (Phospho-Tyr55/53) |
| 118:LIMK1 (Phospho-Thr508) |
| 119:PLCG2 (Phospho-Tyr753) |
| 120:Ras-GRF1 (Phospho-Ser916) |
